# Design and pharmacodynamic study of live biotherapeutic products with efficient degradation of branched‐chain amino acids

**DOI:** 10.1002/btm2.70075

**Published:** 2025-09-15

**Authors:** Zhaowei Chen, Jingyi Xu, Huayue Zhang, Yuezhu Wang, Mingjie Li, Yixiao Wu, Yongqiang Zhu, Yue Liu, Haiyang Xia, Huajun Zheng

**Affiliations:** ^1^ Shanghai‐MOST Key Laboratory of Health and Disease Genomics, NHC Key Lab of Reproduction Regulation, Shanghai Institute for Biomedical and Pharmaceutical Technologies, School of Basic Medical Sciences Fudan University Shanghai China

**Keywords:** branched‐chain amino acids, engineered bacteria, microbiota, synthetic biology

## Abstract

The homeostasis of branched‐chain amino acids (BCAAs) plays a crucial role in maintaining health, and the accumulation of BCAAs can lead to various diseases. Therefore, exogenous degradation or conversion of excessive BCAAs may help alleviate diseases caused by BCAA accumulation, such as maple syrup urine disease. This study utilized synthetic biology approaches to engineer two strains for efficient BCAA catabolism successfully—ECN‐Deg and ECN‐Tra—by integrating specific metabolic pathways into the chassis strain, *Escherichia coli* Nissle 1917 (ECN). ECN‐Deg integrates a metabolic module for BCAA degradation, while ECN‐Tra integrates a metabolic module for BCAA transformation. Both engineered strains demonstrate efficient BCAA catabolism in vitro and in vivo. In a high‐BCAA mouse model, ECN‐Deg and ECN‐Tra alleviated liver and ileal damage caused by excessive BCAAs and reduced systemic inflammation levels. Furthermore, ECN‐Deg and ECN‐Tra were able to modulate the gut microbiota, increasing the richness of *Akkermansia muciniphila* and *Mucispirillum schaedleri*, which are associated with health benefits. Additionally, they reduced the richness of the pathogenic bacterium *Streptococcus pasteurianus*. Thus, this study lays the foundation for the development of probiotics for the treatment of BCAAs metabolic disorders and BCAAs‐related chronic diseases.

**Translational Impact Statement**

This study engineered probiotic bacteria that effectively degrade excess branched‐chain amino acids (BCAAs) in the gut. These live biotherapeutics reduced BCAA‐associated organ damage and inflammation in mice, while beneficially modulating the gut microbiota. This approach offers a promising oral strategy to treat BCAA accumulation disorders like maple syrup urine disease, potentially replacing or reducing the need for highly restrictive diets. It also holds promise for managing chronic diseases linked to BCAA imbalance, such as cardiovascular disease and diabetes.

## 1 INTRODUCTION

Leucine (Leu), isoleucine (Ile), and valine (Val) are called branched‐chain amino acids (BCAAs) because of the methyl group (‐CH3) in their side chains. Adult males typically have a BCAAs (the sum of Leu, Ile, and Val) level of 466.5 (423.6–514.7) μmol/L, whereas adult females typically have a level of 415.3 (382.5–466.0) μmol/L in plasma. The catabolism of BCAAs is essential for maintaining their homeostasis in the body and preventing pathological accumulatio.^1^, ^2^ Excessive BCAAs disrupt glycolysis, fatty acid oxidation, the tricarboxylic acid cycle, and oxidative phosphorylation, leading to mitochondrial dysfunction. BCAAs can directly activate the mTOR signaling pathway, inducing insulin resistance. Additionally, increased BCAAs levels can activate the NF‐κB signaling pathway, upregulating inflammatory signaling and promoting inflammasome formation.^3^ Excessive accumulation of BCAAs can result in various chronic diseases, including cardiovascular diseases, cancer, obesity, and diabetes.^2^, ^4^, ^5^, ^6^, ^7^, ^8^, ^9^, ^10^, ^11^, ^12^ Current interventions mainly rely on dietary restriction of BCAAs or pharmacological approaches such as sodium phenylbutyrate (NaPB), which enhances BCAA catabolism.

In addition, abnormalities in BCAA metabolism are frequently associated with hereditary metabolic disorders. The most common are Maple Syrup Urine Disease, Propionic Acidemia, and Methylmalonic Acidemia, which are caused by mutations in the methylmalonyl CoA mutase (MCM), propionyl‐CoA carboxylase (PCC), and branched‐chain *α*‐ketoacid dehydrogenase complex, respectively.^13^ Their reported incidence rates are 1:280,000, 1:50,000, and 1:139,000.^14^, ^15^, ^16^, ^17^ Upon diagnosis, the primary therapeutic approach is to immediately adjust the child's diet, strictly limiting the intake of BCAAs as well as propionyl‐CoA‐derived amino acids (Ile, Val, Met, and Thr), to reduce the concentration of precursor amino acids in the blood. For patients with severe conditions, in addition to dietary control, comprehensive measures such as pharmacotherapy, hemodialysis, or surgical intervention are necessary.^14^, ^18^, ^19^ However, current intervention strategies remain limited, highlighting the importance of developing new therapeutic approaches to improve treatment outcomes.

*Escherichia coli* Nissle 1917 (ECN) has gained widespread recognition as a safety probiotic and vector strain due to its well‐characterized genetic background, ease of genetic manipulation, and‐most importantly‐its lack of long‐term colonization in healthy humans after oral administration.^20^ It has shown tremendous potential in the fields of disease therapy and drug delivery.^21^, ^22^, ^23^, ^24^ Through synthetic biology approaches, precise “reprogramming” of the chassis strain's genetics simplifies its genetic background while endowing it with specific biological functions, thus creating engineered bacterial live biotherapeutic products (LBPs) with excellent targeting, controllability and safety.^24^ By integrating particular metabolic pathways into the chassis strain, it is possible to effectively absorb and metabolize toxic metabolites within the body. This strategy has shown promising applications in the treatment of various metabolic diseases, such as phenylketonuria,^25^, ^26^ hereditary tyrosinemia type 1,^27^ hyperoxaluria,^28^ hyperuricemia,^29^ and hyperammonemia.^30^ In the Kurtz team's construction of LBPs (SYNB1020) to intervene in hyperammonemia, they knocked out the *argR* gene to maximize arginine synthesis and prevent inhibitory feedback.^30^ Therapeutic strategies based on engineered bacteria have demonstrated great potential in the treatment of a range of diseases.

Therefore, in this study, we aimed to regulate BCAAs metabolism to mitigate damage caused by excessive BCAAs accumulation. Through comprehensive literature mining and experimental validation, we constructed two high‐efficiency BCAAs metabolic pathways: the Degradation module (Deg module) and the Transformation module (Tra module). The Deg module was designed to achieve complete BCAA catabolism by enhancing cellular uptake (via the native transporter BrnQ),^31^, ^32^, ^33^ decarboxylating key intermediates (using a heterologous ketoacid decarboxylase from *Lactococcus lactis*, KdcA),^34^, ^35^, ^36^ and amplifying endogenous propionyl‐CoA catabolism (by overexpressing the transcriptional activator PrpR to drive the native 2‐methylcitric acid cycle).^37^, ^38^, ^39^, ^40^ In parallel, the Tra module was engineered to directly convert BCAAs into short‐chain fatty acids (SCFAs) by combining BrnQ‐mediated uptake with Por (a pyruvate flavodoxin/ferredoxin oxidoreductase from *Clostridium sporogenes*) capable of transforming BCAAs into corresponding SCFAs. Several studies have confirmed that this protein is capable of converting BCAAs into the corresponding SCFAs.^41^, ^42^, ^43^, ^44^ On the other hand, considering endogenous BCAAs biosynthesis in ECN, we further knocked out genes associated with BCAAs synthesis pathways, substantially enhancing the engineered strains' BCAAs metabolic capacity. Subsequent screening under in vitro conditions and simulated intestinal fluid (SIF) environments identified the optimal engineered ECN strain with superior BCAAs catabolic performance. Using a high‐BCAAs murine model, we demonstrated that the engineered ECN effectively metabolized excess BCAAs in vivo while alleviating associated pathological injuries. Notably, the engineered ECN exhibited microbiota‐modulating capabilities by increasing intestinal richness of bacteria associated with health benefits and suppressing harmful species, thereby ameliorating gut dysfunction. Given the remarkable therapeutic efficacy demonstrated by our engineered strain, this work establishes a novel paradigm for developing engineered bacterial therapeutics and provides critical insights for advancing microbiome‐based intervention strategies.

## 2 RESULTS

### 2.1 The design of Deg module and Tra module

**FIGURE 1:**
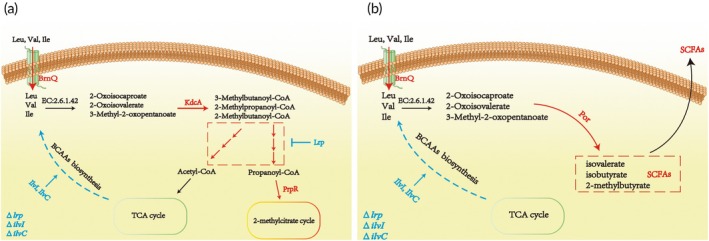
Metabolic module design of two types of LBPs. (a) Design of the “BCAAs *De Novo* Degradation (Deg Module)” metabolic module. (b) Design of the “SCFA Transformation (Tra Module)” metabolic module. In the figure, E2.6.1.42 is branched‐chain amino acid aminotransferase, which is responsible for the transamination of BCAAs to the corresponding *α*‐keto acids, which are metabolized by heterologously expressed KdcA and por. The red arrows indicate the enhanced reaction, and the blue arrows indicate the suppressed reaction. BrnQ, branched‐chain amino acid transporter; KdcA, branched‐chain *α*‐keto acid decarboxylase; PrpR, propionate catabolism operon regulatory protein; Por, pyruvate flavodoxin/ferredoxin oxidoreductase; *lrp*, leucine‐responsive regulatory protein; *ilvI*, acetolactate synthase/acetohydroxybutanoate synthase, catalytic subunit; *ilvC*, ketol‐acid reductoisomerase (NADP(+)).

In this study, we engineered two metabolic modules using ECN as the chassis strain, employing distinct strategies to efficiently catabolize BCAAs: (1) The “BCAAs *De Novo* Degradation (Deg Module)” (Figure 1a): This module incorporates the ECN‐specific branched‐chain amino acid transporter (BrnQ), which enhances the uptake efficiency of BCAAs by ECN. Next, we introduce branched‐chain *α*‐keto acid decarboxylase (KdcA) from *L. lactis*, a protein that has been shown to possess strong dehydrogenase‐decarboxylase activity for BCKAs. Subsequently, to accelerate propionyl‐CoA catabolism—a key intermediate from BCAA degradation—we overexpressed the endogenous transcriptional activator PrpR. This amplifies the native 2‐methylcitric acid cycle (PrpBCDE operon) in ECN, enhancing the downstream metabolic processes of BCAAs. (2) The “BCAAs Transformation (Tra Module)” (Figure 1b): This module is designed around the pyruvate flavodoxin/ferredoxin oxidoreductase (Por) from *C. sporogenes*. Therefore, this module enhances BCAAs uptake through BrnQ and subsequently converts BCAAs into SCFAs via Por. Additionally, we knocked out genes related to the BCAAs biosynthesis pathway in ECN to further increase its demand for BCAAs.

### 2.2 Preparation and characterization of Deg module and Tra module

**FIGURE 2:**
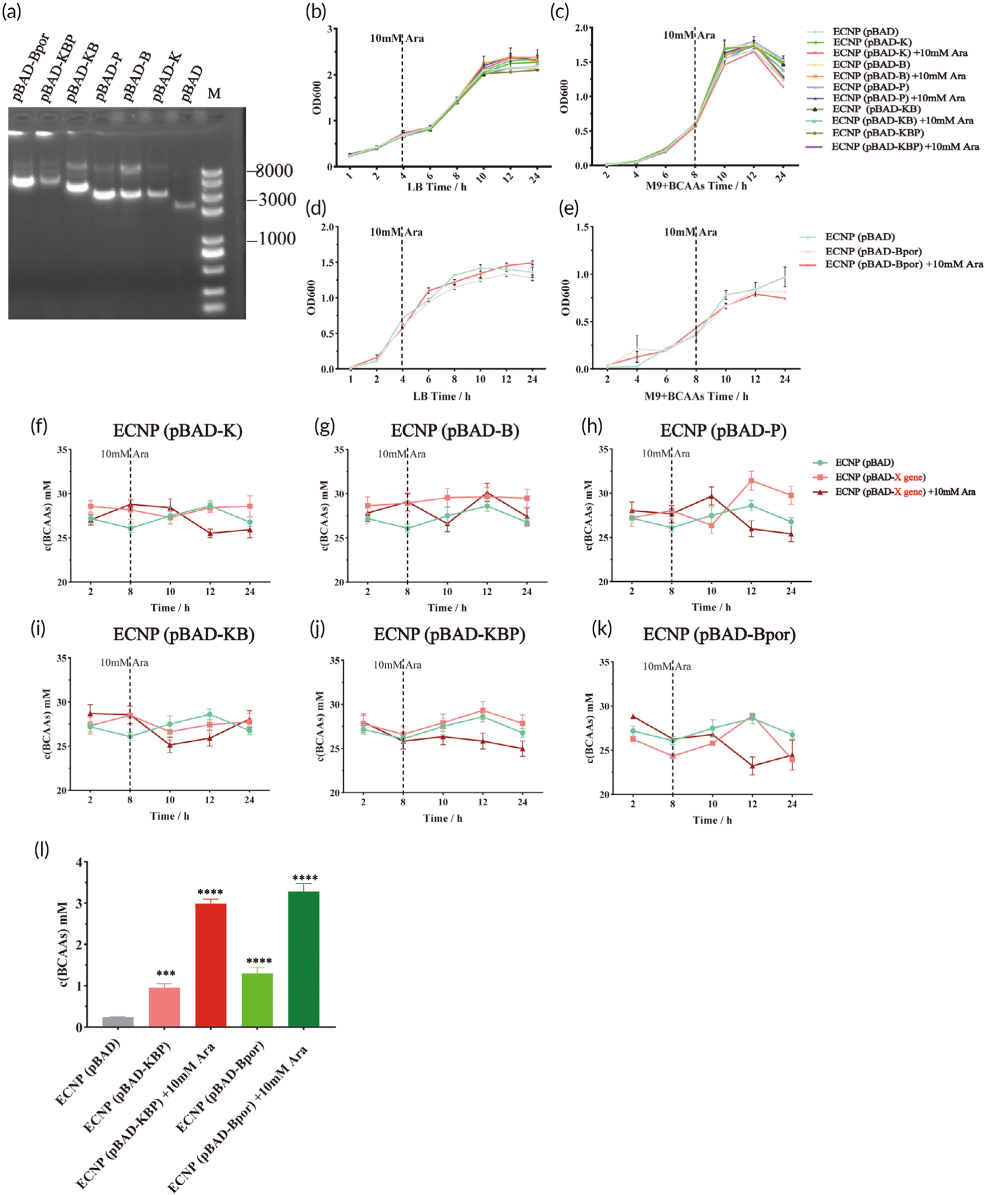
Growth curves and BCAAs degradation capacity of Single/Multiple gene strains. (a) Electrophoresis results of single and multiple gene plasmids. (b), (c) Growth conditions of single/multiple gene clones in the “Deg Module” under LB (b)/M9 + BCAAs (c) conditions. (d), (e) Growth conditions of multiple gene clones in the “Tra Module” under LB (d)/M9 + BCAAs (e) conditions. (f)–(k) BCAAs concentrations in the supernatant at different time points in M9 + 30 mM BCAAs medium for each engineered strain; Among them, “X‐gene” represents the general term of the target genes carried by the corresponding strains. For example, the “X‐gene” of ECNP (pBAD‐K) refers to *kcdA*, while the “X‐gene” of ECNP (pBAD‐KBP) refers to *kcdA*, *brnQ*, and *prpR*. (l) Total BCAAs consumption by engineered strains within 24 h. pBAD‐K: PBAD‐*kcdA*; pBAD‐B: PBAD‐*brnQ*; pBAD‐P: PBAD‐*prpR*; pBAD‐KB: PBAD‐*kcdA*‐*brnQ*; pBAD‐KBP: PBAD‐*kcdA*‐*brnQ*‐*prpR*; pBAD‐Bpor: PBAD‐*brnQ*‐*por*. Statistical analysis was performed using one‐way ANOVA (*p <0.05, **p <0.01, ***p <0.001, ****p <0.0001). Data are represented as mean ± SEM.

To evaluate the effects of different genes on the growth of the chassis strain and its ability to degrade BCAAs, we first constructed plasmids containing single and multiple target genes, including pBAD‐*kcdA* (pBAD‐K), pBAD‐*brnQ* (pBAD‐B), pBAD‐*prpR* (pBAD‐P), pBAD‐*kcdA*‐*brnQ* (pBAD‐KB), pBAD‐*kcdA*‐*brnQ*‐*prpR* (pBAD‐KBP), and pBAD‐*brnQ*‐*por* (pBAD‐Bpor) (Figure 2a). To ensure stable inheritance of the recombinant pBAD plasmids (ColE1 origin of replication), we first cured two cryptic endogenous plasmids (pMUT1 and pMUT2) from ECN due to their incompatible ColE1‐type replication origins. These engineered plasmids were then transformed into the resulting plasmid‐free chassis strain (designated ECNP), and their effects on bacterial growth were evaluated under different conditions (Figure 2b–e). The results showed no significant differences in growth among the single‐gene and multi‐gene strains in either LB medium or M9 + 30 mM BCAAs medium. However, there were notable differences in BCAAs catabolism among the strains (Figure 2f–k). Induced by 10 mM Ara, the BCAAs degradation capacity of all engineered strains was enhanced. However, a majority of strains exhibited some degree of metabolic disruption. Specifically, we hypothesized that ECNP (pBAD‐B) rapidly takes up a large amount of BCAAs post‐induction (8–10 h), but due to its limited catabolic capacity, fails to effectively utilize them, leading to subsequent expulsion (10–12 h) (Figure 2g). Compared with ECNP (pBAD‐B), BCAAs in the medium of ECNP (pBAD‐KB) did not fluctuate significantly, but increased slowly at 10–24 h. We speculate that this is due to the fact that ECNP (pBAD‐KB) intake of BCAAs is greater than the ability to metabolize BCAAs, resulting in incomplete metabolism of BCAAs and slow excretion (Figure 2i). In conclusion, none of these strains can stably degrade BCAAs. Only ECNP (pBAD‐KBP) and ECNP (pBAD‐Bpor) demonstrated more stable BCAAs consumption capabilities and utilized the highest total amounts of BCAAs within 24 h, 2.983 ± 0.110 mM and 3.281 ± 0.193 mM, respectively (Figure 2l). We therefore propose that the combination of *kcdA*, *brnQ*, and *prpR*, as well as the combination of *brnQ* and *por*, form more efficient BCAAs catabolic modules in the chassis strain.

**FIGURE 3:**
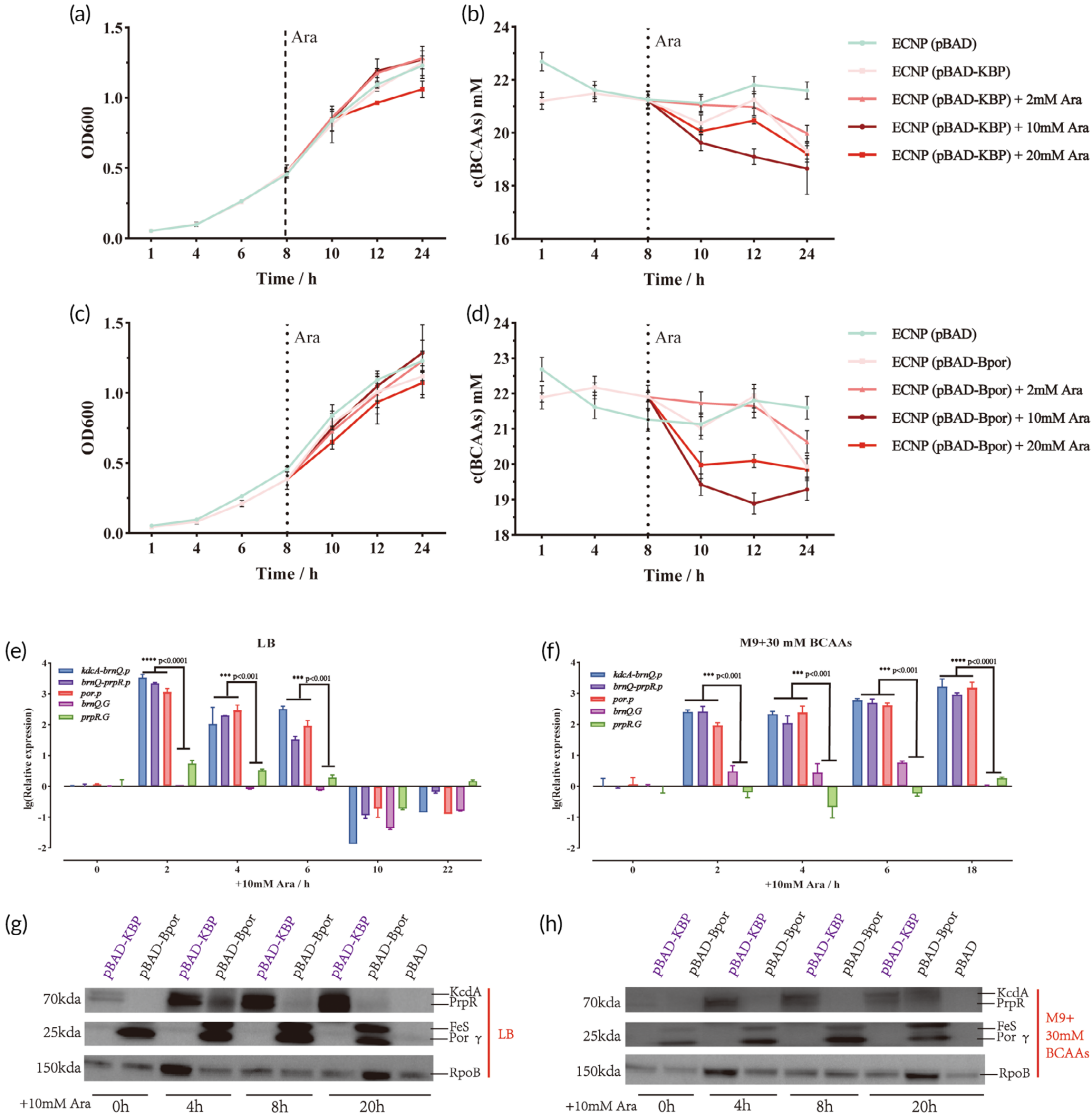
Induction effects of 10 mM Ara. (a), (b) Growth condition (a) and BCAAs content (b) of ECNP (pBAD‐KBP)in M9 + 30 mM BCAAs with different Ara concentrations (0, 2, 10, 20 mM). (c), (d) Growth condition (c) and BCAAs content (d) of ECNP (pBAD‐Bpor) in M9 + 30 mM BCAAs with different Ara concentrations (0, 2, 10, 20 mM). (e), (f): MRNA induction effects of 10 mM Ara on target genes in LB (e) and M9 + 30 mM BCAAs (f) media (*x*‐axis: Lg(Relative expression), *y*‐axis: +10 mM Ara/h). The *rpoA* gene was used as a control reference. (g), (h) Protein induction effects of 10 mM Ara on target proteins in LB (g) and M9 + 30 mM BCAAs (h) media (supernatant proteins). Recombinant anti‐RNA polymerase beta (RpoB) serves as a loading control to verify consistent protein loading across samples. pBAD‐KBP: PBAD‐*kcdA*‐*brnQ*‐*prpR*; pBAD‐Bpor: PBAD‐*brnQ*‐*por*. *kcdA‐brnQ.p*: mRNA expression levels of *kcdA* and *brnQ* on the plasmid; *brnQ‐prpR.p*: mRNA expression levels of *brnQ* and *prpR* on the plasmid; *por.p*: mRNA expression levels of *por* on the plasmid; *brnQ.G*: mRNA expression levels of *brnQ* on the genome; *prpR.G*: mRNA expression levels of *prpR* on the genome; Por *γ*: *γ* subunit of the Por complex. Statistical analysis was performed using one‐way ANOVA (*p <0.05, **p <0.01, ***p <0.001, ****p <0.0001). Data are represented as mean ± SEM.

After confirming that two modules exhibited BCAAs degradation efficacy, we explored the induction threshold of Ara. First, we examined the effects of 2, 10, and 20 mM Ara on the strains' growth and BCAAs degradation capacity. As shown in Figure 3a,c, 20 mM Ara slightly inhibited the growth of ENCP (pBAD‐KBP) and ENCP (pBAD‐Bpor), while 2 mM and 10 mM Ara did not significantly affect the strains' growth. Regarding BCAAs degradation, the strain exhibited the highest BCAAs consumption (24 h) under 10 mM Ara induction (Figure 3b,d). Additionally, we investigated the molecular‐level effects of 10 mM Ara using quantitative real‐time PCR (qPCR) and Western blot (WB) analyses. Since *brnQ* and *prpR* are endogenous genes present in the ECNP genome, we first designed qPCR primers to distinguish between plasmid‐derived and genome‐derived expression of the target genes (Table S5). In LB medium, transcriptional levels of the target gene rapidly peaked at 3970‐fold within 2 h following induction with 10 mM Ara, but subsequently declined over time. In contrast, under M9 medium supplemented with 30 mM BCAAs, plasmid‐borne gene expression exhibited a gradual upregulation pattern, reaching 2443‐fold post‐induction within 2 h (Figure 3e,f). To investigate the expression of the target genes at the protein level, we appended a His tag to the C‐terminus of each target protein. The results showed that the target proteins were successfully expressed in both LB and M9 + 30 mM BCAAs media, with minor leaky expression observed before the addition of the inducer (Figure 3g,h).

### 2.3 Knockout of BCAAs biosynthesis pathway genes ilvC, ilvI, and lrp

To disrupt endogenous BCAAs production in ECN, we engineered three sgRNA‐containing plasmids for CRISPR‐Cas9‐mediated knockout of the BCAAs biosynthetic genes *ilvC*, *ilvI*, and the transcriptional regulator *lrp* (Figure S1A). *ilvC* and *ilvI* genes encode ketol‐acid reductoisomerase and the catalytic subunit of acetolactate synthase/acetohydroxybutanoate synthase, respectively— both key enzymes in the BCAAs anabolism of the strain. Therefore, the knockout of these genes disrupts the strain's ability to synthesize BCAAs. Using the constructed guide plasmids, we successfully knocked out *ilvC* and *ilvI*, resulting in the strains ECNPΔ*ilvI*, ECNPΔ*ilvC*, and ECNPΔ*ilvI*Δ*ilvC* (Figure S1B–E). *lrp* encodes the leucine‐responsive regulatory protein, a transcription factor that activates BCAAs biosynthesis while inhibiting BCAAs catabolism. By knocking out this gene using guide plasmids, we generated the strains ECNPΔ*lrp*, ECNPΔ*ilvI*Δ*lrp*, ECNPΔ*ilvC*Δ*lrp*, and ECNPΔ*ilvI*Δ*ilvC*Δ*lrp*, thereby further enhancing the chassis strain's ability to utilize BCAAs (Figure S1F,G). By blocking the endogenous BCAAs biosynthesis pathway, we enhance chassis dependency on exogenous BCAAs consumption. Upon depletion of exogenous BCAAs, the chassis ceases proliferation and subsequently undergoes self‐elimination from the intestinal tract.

### 2.4 Identification of the efficient BCAAs‐metabolizing engineered strains (ECN‐Deg and ECN‐Tra)

**FIGURE 4:**
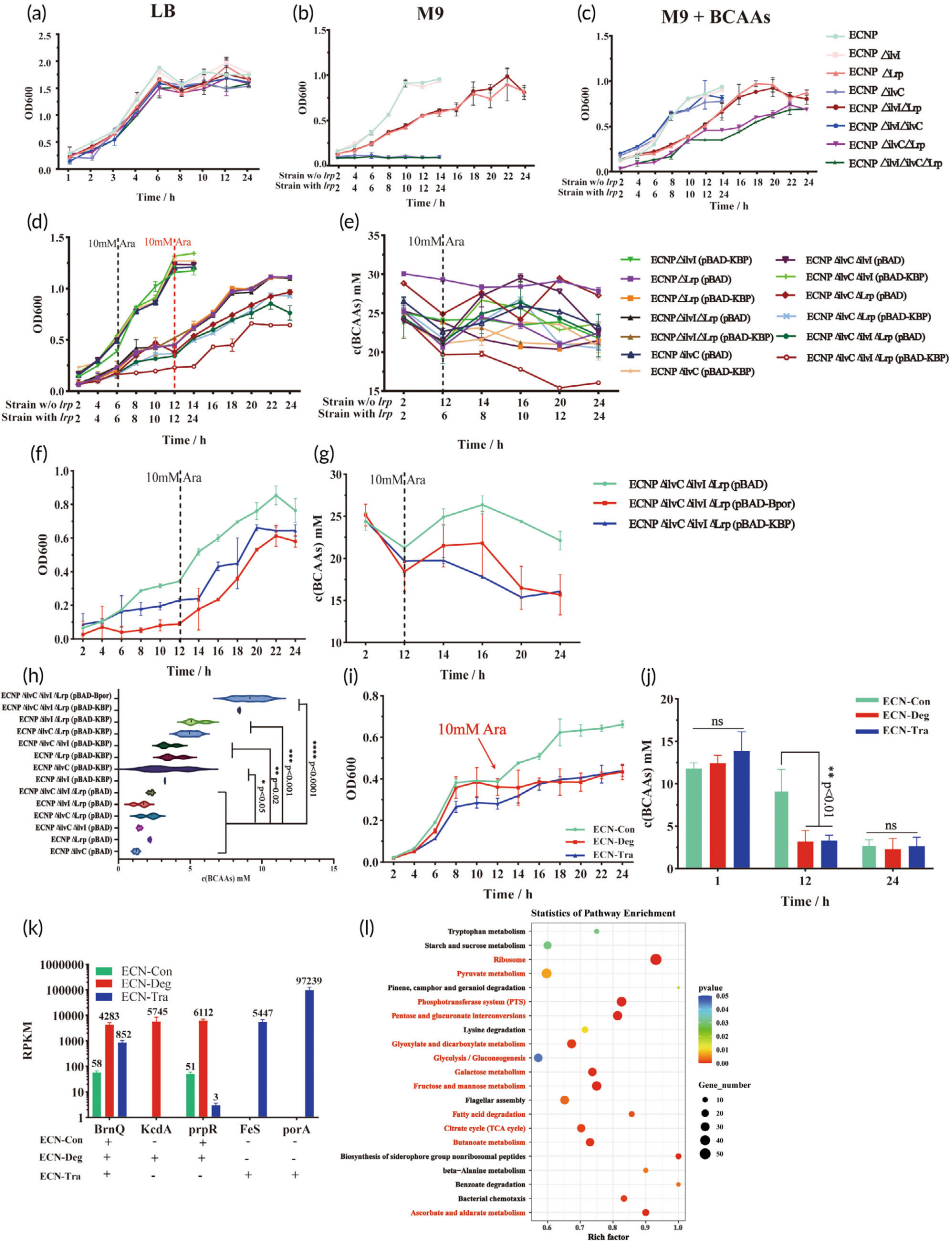
Growth condition and BCAAs degradation capacity of engineered strains. (a)–(c) Growth condition of various genetic knockout strains in LB (a), M9 (b), and M9 + BCAAs (c) media. (d)–(e) Growth condition (d) and BCAAs degradation capacity (e) of “Deg Module” engineered strains in M9 + 30 mM BCAAs medium. In (b)–(e), the *X*‐axis “Strain w/o *lrp*” represents that these strains lack the *lrp* gene, while “Strain with *lrp*” represents that these strains contain the *lrp* gene. (f), (g) Comparison of growth condition (f) and BCAAs degradation capacity (g) between “Deg Module” and “Tra Module” engineered strains in M9 + 30 mM BCAAs medium. (h) Total BCAAs consumed by each engineered strain within 24 h. (i), (j) The growth condition (i) and BCAAs degradation capacity (j) of ECN‐Con, ECN‐Deg, and ECN‐Tra in SIF. (k) The expression levels of each target gene when the engineered bacteria were cultured in SIF to the end point of the logarithmic growth phase (8 h). (l) KEGG enrichment analysis of common upregulated differentially expressed genes in ECN‐Deg and ECN‐Tra versus ECN‐Con at 8 h. ECN‐Con: ECNP*ΔilvIΔilvCΔlrp* (pBAD), ECN‐Deg: ECNP*ΔilvIΔilvCΔlrp* (pBAD‐KBP), ECN‐Tra: ECNP*ΔilvIΔilvCΔlrp* (pBAD‐Bpor). Statistical analysis was performed using one‐way ANOVA (*p <0.05, **p <0.01, ***p <0.001, ****p <0.0001). Data are represented as mean ± SEM.

After constructing a series of knockout strains, we evaluated their growth profiles to assess their dependency on exogenous BCAAs, thereby screening for suitable chassis strains (Figure 4a–c). In LB, no significant differences were observed in the growth of various knockout strains. In M9, the Δ*ilvC* strains failed to grow, the Δ*lrp* strains showed slow growth, and the Δ*ilvI* strains grew normally. In M9 + 30 mM BCAAs, the Δ*lrp* strains showed reduced growth rates, and strains with simultaneous deletions of Δ*lrp* and Δ*ilvC* exhibited further reduced growth, whereas the Δ*ilvI* strains maintained normal growth. These findings indicate that *ilvC* is a critical gene for BCAAs biosynthesis, as its absence prevents ECN from synthesizing BCAAs de novo, making it reliant on exogenous BCAAs for survival. The deletion of *lrp*, which encodes a leucine‐responsive regulatory protein involved in the regulation of amino acid anabolism and catabolism, significantly impaired the strain's ability to adapt to its environment and regulate metabolism, thereby affecting growth.^45^, ^46^ After characterizing the growth behavior of the knockout strains, we introduced the plasmids pBAD, pBAD‐KBP, and pBAD‐Bpor into the knockout strains and assessed their growth and BCAAs degradation capacities in M9 + 30 mM BCAAs. We found that strains ECNP*ΔilvIΔilvCΔlrp* (pBAD‐KBP) and ECNP*ΔilvIΔilvCΔlrp* (pBAD‐Bpor) exhibited reduced growth rates, while the BCAAs concentrations in the supernatant decreased over time (Figure 4d–g). Regarding the total BCAAs consumption within 24 h, the engineered strains harboring pBAD‐KBP and pBAD‐Bpor consumed significantly more BCAAs than the control strain harboring pBAD. Notably, ECNP*ΔilvIΔilvCΔlrp* (pBAD‐KBP) and ECNP*ΔilvIΔilvCΔlrp* (pBAD‐Bpor) exhibited the highest degradation capacities, consuming 8.414 ± 0.051 mM and 9.146 ± 1.019 mM BCAAs, respectively, within 24 h (Figure 4h). Ultimately, we named the two most efficient BCAAs‐metabolizing engineered strains as ECN‐Deg for ECNP*ΔilvIΔilvCΔlrp* (pBAD‐KBP) and ECN‐Tra for ECNP*ΔilvIΔilvCΔlrp* (pBAD‐Bpor). The strain ECNP*ΔilvIΔilvCΔlrp* (pBAD) was named ECN‐Con, which served as the control strain for subsequent experiments.

We further investigate the BCAAs degradation capacity of ECN‐Deg and ECN‐Tra in SIF. ECN‐Deg and ECN‐Tra rapidly consume BCAAs to a relatively low level that only provides the survival of engineered strains instead of proliferation within 8 h (Figure 4i,j). When adding the additional carbohydrate to SIF, ECN‐Deg and ECN‐Tra do not return to the logarithmic stage, but ECN‐Con does (Figure 4i). Measurement of BCAAs content in SIF at 12 h revealed that ECN‐Deg and ECN‐Tra consumed the majority of BCAAs. Consequently, protein synthesis was impaired, ultimately leading to the cessation of proliferation in the engineered strains (Figure 4j). On the other hand, we find the core genes in the target metabolic pathways carried by ECN‐Deg and ECN‐Tra exhibited high levels of expression in the SIF environment (Figure 4k). Further comparison of differentially expressed genes (DEGs) between ECN‐Con and ECN‐Deg/ECN‐Tra revealed that the upregulated DEGs in ECN‐Deg and ECN‐Tra were significantly enriched in energy metabolism‐related pathways, such as the Ribosome and TCA cycle (Figure 4l). This suggests that efficient degradation of BCAAs and overexpression of the target protein may require additional energy supply, leading to significant changes in the metabolic pathways of the engineered bacteria.

### 2.5 Investigating gut retention of ECN‐Deg and ECN‐Tra in mice

**FIGURE 5:**
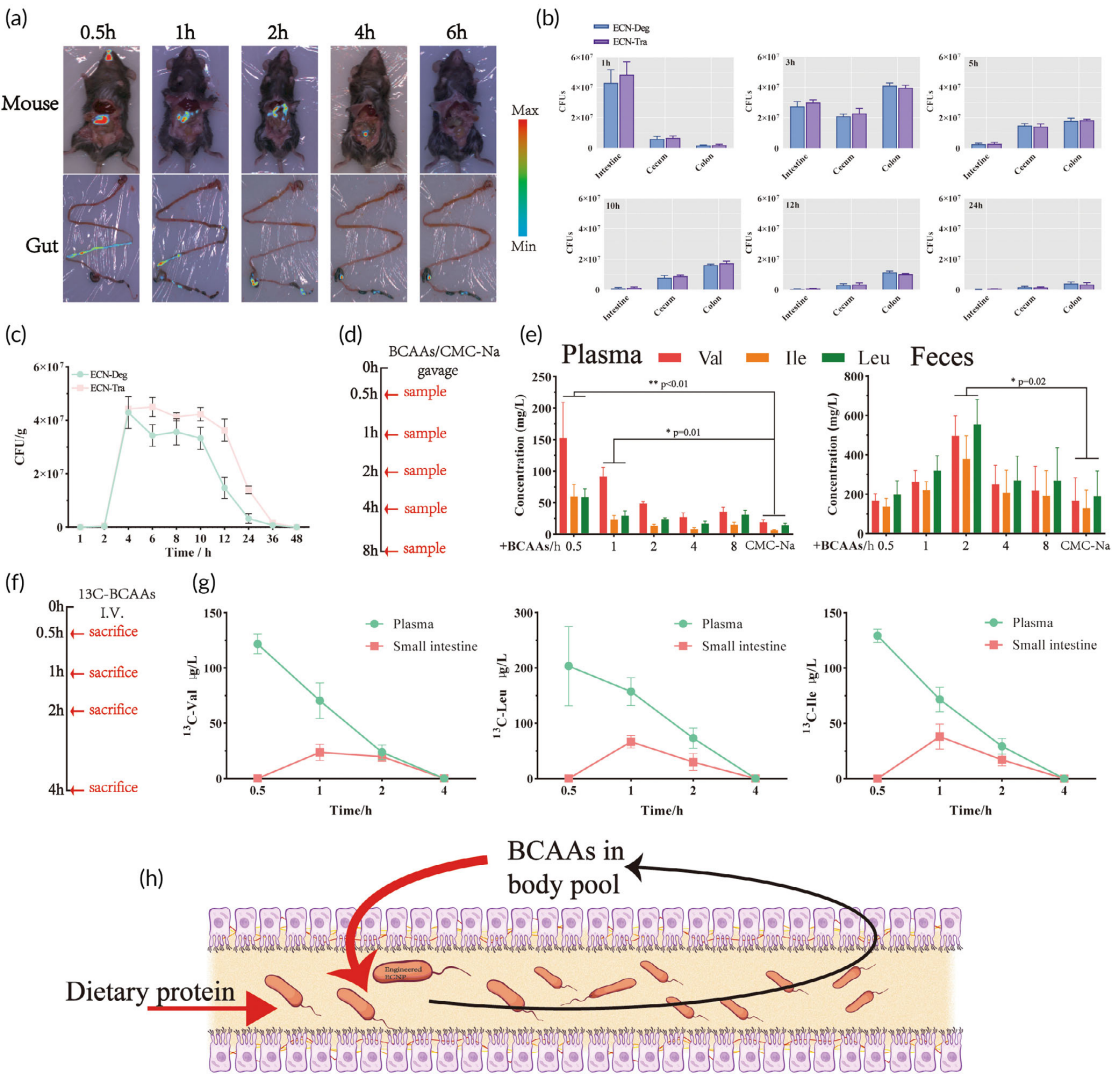
Investigating gut retention of ECN‐Deg and ECN‐Tra and basal metabolism and intestinal‐blood circulation of BCAAs. (a) Bioluminescent images of mice and their gastrointestinal tract at different time points following gavage of ECN‐Lux. (b) Quantification of viable bacteria in the intestine (jejunum + ileum), colon, and cecum at 1, 3, 5, 10, 12, and 24 h after gavage of ECN‐Deg and ECN‐Tra. (c) Quantitative analysis of viable bacteria in feces at different time points after gavage of ECN‐Deg and ECN‐Tra. (d) Experimental procedure for exploring the basal metabolism of BCAAs in C57BL/6 mice. (e) Basal metabolism of BCAAs in C57BL/6 mice after gavage of BCAAs (200 μg/g BCAAs). (f) Experimental procedure for verifying BCAAs intestinal‐blood circulation. (g) Intravenous injection of ^13^C‐BCAAs (20 μg/g BCAAs) in C57BL/6 mice. Mice were sacrificed at specified time points to measure the concentration of ^13^C‐BCAAs in plasma and intestinal effluent. (h) BCAAs enter the intestine through the intestinal‐blood circulation and are degraded by the engineered bacteria. CMC‐Na: sodium carboxymethyl cellulose. Statistical analysis was performed using one‐way ANOVA (*p <0.05, **p < 0.01, ***p <0.001, ****p <0.0001). Data are represented as mean ± SEM.

To gain deeper insights into the metabolic kinetics of the two engineered strains, we constructed a reporter plasmid expressing luciferase (pBAD‐*luxCDABE*) and introduced it into the chassis strain ECNPΔ*ilvI*Δ*ilvC*Δ*lrp*, generating a luciferase‐expressing engineered strain, named ECN‐Lux, for subsequent in vivo tracking experiments. The results revealed that, within 0.5 h, ECN‐Lux rapidly traversed the stomach and duodenum, reaching the jejunum. After 1 h, the strain accumulated primarily in the ileum. Another 4 h later, ECN‐Lux had moved to the cecum, where it eventually formed feces, entering the colon for excretion (Figure 5a).

Additionally, the mice were divided into two groups, and each group was gavaged with 2 × 10^10^ CFU of different engineered strains (ECN‐Deg, and ECN‐Tra). We explored the distribution of the engineered strains across various regions of the gut. Within 3 h, the strains were primarily localized to the small intestine, and their richness in this region gradually decreased over time. The retention times of ECN‐Deg and ECN‐Tra in the cecum and colon were considerably longer than in the small intestine (Figure 5b). Fecal samples were collected at different time points to quantify the bacterial load, providing insights into the colonization time of ECN‐Deg and ECN‐Tra in the mouse gut. As shown in Figure 5c, the engineered strains were rapidly excreted with the feces after 4 h of gavage, and only small amounts remained in the feces up to 48 h post‐gavage, with the bacterial counts at this time point being (2.0 ± 0.2) × 10^4^ and (4.64 ± 0.305) × 10^4^ for ECN‐Deg and ECN‐Tra, respectively. The results indicated that the engineered strains retained in the gut for approximately 48 h.

### 2.6 Basal metabolism and intestinal‐blood circulation of BCAAs in C57BL/6 mice

To further investigate whether ECN‐Deg and ECN‐Tra can efficiently degrade BCAAs in vivo, we first examined the basal metabolic rate of BCAAs in C57BL/6 mice. Mice were gavaged with 200 μg/g BCAAs or an equal volume of 0.5% CMC‐Na (Sodium carboxymethyl cellulose) solution. Blood plasma and feces were then collected at specific time points for BCAAs qualification (Figure 5d). The results showed that 0.5 h after the gavage, a large number of BCAAs were absorbed by the intestine and entered the bloodstream, and the BCAAs were degraded to normal levels within 2 h (Figure 5e). This timing precisely overlaps with the intestinal retention period of the engineered strains, indicating that oral administration of the strains immediately following exogenous BCAAs intake may enable them to efficiently utilize the supplemented BCAAs during the small intestinal phase.

To further explore the bidirectional flux of BCAAs between the intestinal lumen and bloodstream, we intravenously injected (I.V.) ^13^C‐labeled BCAAs (20 μg/g BCAAs) and measured the levels of ^13^C‐BCAAs in the plasma and intestinal lumen at specific time points (Figure 5f). The results showed that over time, the concentration of ^13^C‐BCAAs in the plasma rapidly decreased. Approximately 1 h after intravenous injection, a portion of the ^13^C‐BCAAs in the plasma entered the intestinal lumen, and by 4 h, the ^13^C‐BCAAs in both the plasma and intestinal lumen were completely absorbed by the body (Figure 5g). This experiment confirmed the presence of intestinal‐blood circulation of BCAAs, providing an additional opportunity for the intestine‐resident engineered bacteria to utilize BCAAs (Figure 5h).

### 2.7 Efficient BCAAs catabolism of ECN‐Deg and ECN‐Tra in mice

**FIGURE 6:**
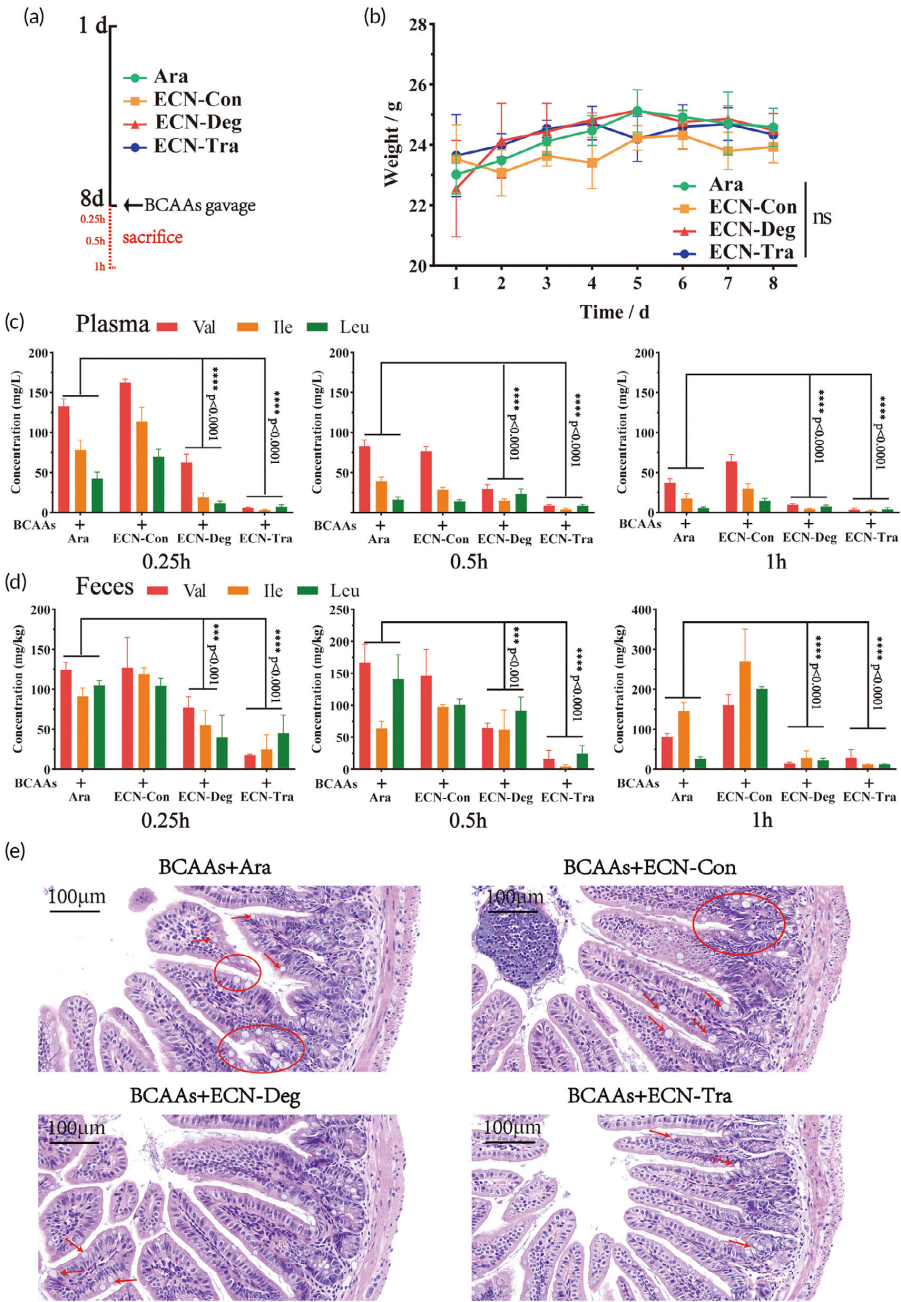
Efficient BCAAs Catabolism of ECN‐Deg and ECN‐Tra in Mice. (a) Experimental scheme for the acute high‐BCAAs mouse model. (b) Body weight changes in mice after 8 days of continuous engineered bacteria intervention. (c) Changes in BCAAs levels in plasma at different time points after gavage of BCAAs (200 μg/g BCAAs) in different treatment groups. (d) Changes in BCAAs levels in feces at different time points after gavage of BCAAs in different treatment groups. (e) H&E staining of ileum sections from different treatment groups 0.5 h after gavage of BCAAs. Statistical analysis was performed using one‐way ANOVA (*p <0.05, **p <0.01, ***p <0.001, ****p <0.0001). Data are represented as mean ± SEM.

To assess whether ECN‐Deg and ECN‐Tra could efficiently metabolize BCAAs in vivo, we next evaluated their ability to reduce BCAAs levels in an acute high‐BCAAs mouse model. To ensure maximum efficacy of the engineered bacteria, wild‐type mice in different groups were orally administered 2 × 10^10^ CFU of ECN‐Con, ECN‐Deg, or ECN‐Tra once daily for 8 consecutive days. On day 8, after gavage of high BCAAs, plasma and fecal samples were collected at 0.25, 0.5, and 1 h after BCAAs gavage to measure BCAAs levels (Figure 6a). Ara was used as a blank control in this experiment, and ECN‐Con served as a negative control. The results showed that continuous, high‐dose intervention with engineered bacteria did not affect the mice's body weight, indicating good tolerance to the strains (Figure 6b). Regarding BCAAs content, both ECN‐Deg and ECN‐Tra, especially ECN‐Tra, exhibited strong BCAAs consumption capability. In the mice receiving ECN‐Deg and ECN‐Tra, BCAAs levels in both plasma and feces were significantly reduced (Figure 6c,d).

Additionally, excessive BCAAs intake caused acute inflammatory damage in the ileum, with mild villus damage and numerous inflammatory vacuoles observed. However, the mice treated with ECN‐Deg and ECN‐Tra showed reduced inflammation in the ileum, with more organized and densely packed villi (Figure 6e). This experiment, conducted in an acute high‐BCAAs treatment mouse model, demonstrates that ECN‐Deg and ECN‐Tra can consume excess BCAAs in the intestine and protect against ileal injury.

### 2.8 ECN‐Deg and ECN‐Tra alleviate damage induced by BCAAs

**FIGURE 7:**
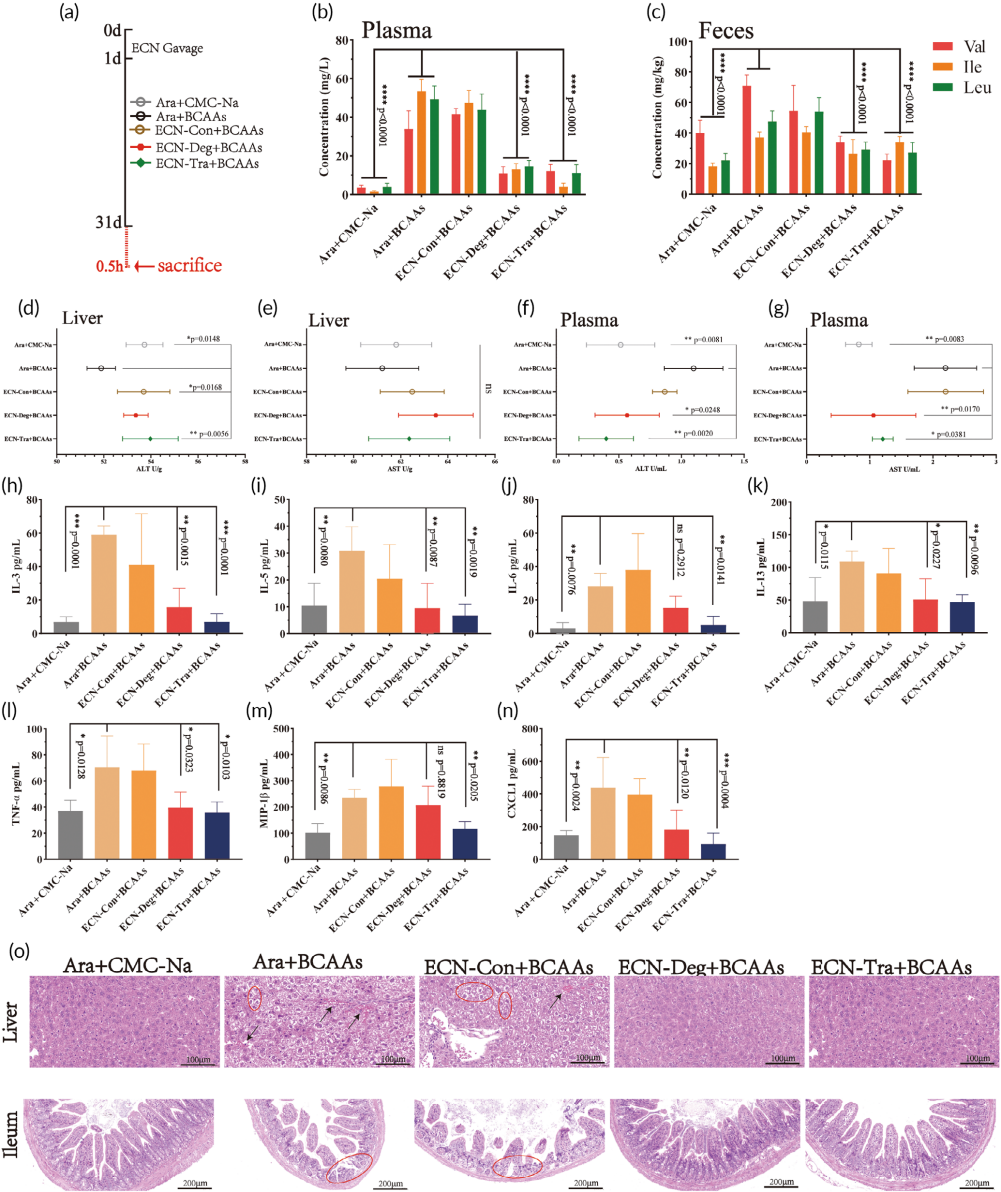
The therapeutic effect of ECN‐Deg and ECN‐Tra against BCAAs‐induced damage in mice. (a) Experiment schedule for the 30‐dayperiods high‐BCAAs mouse model. (b), (c) The qualification of BCAAs in plasma (b) and feces (c). (d), (e) The qualification of ALT (d) and AST (e) in the liver. (f), (g) The qualification of ALT (f) and AST (g) in plasma. (h)–(n) The concentration of IL‐3 (h), IL‐5 (i), IL‐6 (j), IL‐13 (k), TNF‐*α* (l), MIP‐1*β* (m), and CXCL1 (n) in plasma. (o) H&E staining of liver and ileum sections from different treatment groups. CMC‐Na: sodium carboxymethyl cellulose. Statistical analysis was performed using one‐way ANOVA (*p <0.05, **p <0.01, ***p <0.001, ****p <0.0001). Data are represented as mean ± SEM.

After confirming the efficacy of BCAAs degradation mediated by these two engineered strains in the acute high‐BCAAs mouse model, we next investigated whether ECN‐Deg and ECN‐Tra could alleviate the phenotype of disease induced by BCAAs, further evaluating the clinical potential of these strains. In this experiment, mice were randomly divided into five groups for different interventions. Except for the Ara + CMC‐Na group, which served as a blank control without additional BCAAs during the 30‐day experiment, the other four groups were orally administered high‐concentration BCAAs (200 μg/g BCAAs) daily, along with the corresponding engineered bacteria (2 × 10^10^ CFU) or an equivalent volume of 10 g/L Ara (Figure 7a). Mice treated with ECN‐Deg and ECN‐Tra significantly reduced BCAAs concentrations in both plasma and feces, while the mice treated with ECN‐Con exhibited BCAAs levels in plasma and feces similar to those of the Ara + BCAAs treated group (Figure 7b,c). Moreover, BCAAs treatment exacerbated the liver's ammonia detoxification burden, leading to liver cell damage and the leakage of ALT and AST into the plasma, resulting in a significant increase in ALT and AST enzyme activities. In contrast, intervention with ECN‐Deg and ECN‐Tra significantly reduced plasma ALT and AST levels (Figure 7d–g). In addition, excessive BCAAs aggravated systemic inflammation, as evidenced by a significant increase in inflammatory cytokines such as interleukin‐3 (IL‐3), interleukin‐5 (IL‐5), interleukin‐6 (IL‐6), interleukin‐13 (IL‐13), tumor necrosis factor *α* (TNF‐*α*), macrophage inflammatory protein *β* (MIP‐1*β*), and chemokine (C‐X‐C motif) ligand 1 (CXCL1) (Figure 7h–n). However, ECN‐Deg and ECN‐Tra effectively alleviated the inflammation by efficiently utilizing excess BCAAs, maintaining the cytokine levels at normal levels. Histological analysis of liver and ileum tissues showed that excessive BCAAs induced widespread ballooning degeneration in the liver, accompanied by focal inflammatory reactions. After treatment with the two engineered strains, the degree of liver damage and inflammation was significantly reduced. Furthermore, excessive BCAAs also caused villus atrophy and disrupted circular fold arrangement in the ileum, along with notable inflammatory cell infiltration. However, ECN‐Deg and ECN‐Tra effectively alleviated ileal damage (Figure 7o). These results suggest that ECN‐Deg and ECN‐Tra enhance BCAAs degradation in mice, reduce the accumulation of toxic metabolites, and alleviate the resulting organ damage.

### 2.9 Regulatory effect of ECN‐Deg and ECN‐Tra on gut microbiota

**FIGURE 8:**
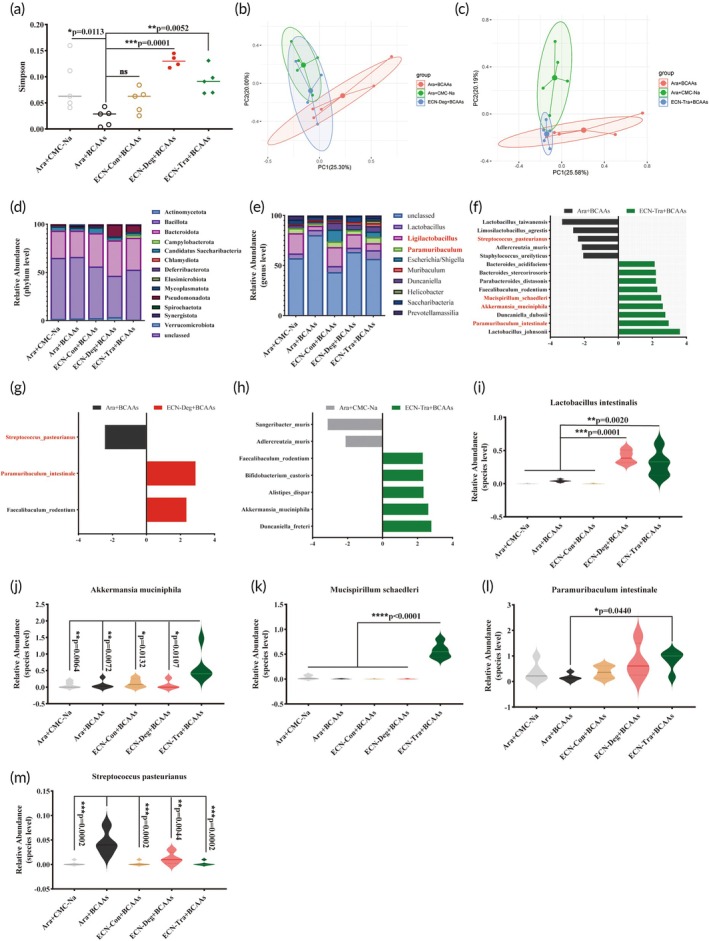
ECN‐Deg and ECN‐Tra modulate gut microbiota in the BCAAs model in the high‐BCAAs mouse model. (a) Effects of ECN‐Deg and ECN‐Tra on the Simpson index of gut microbiota. (b), (c) PCoA analysis of gut microbiota. (d), (e) Microbial taxa composition analysis at the phylum level (d) and the genus level (e). (f)–(h) LDA analysis in Ara + BCAAs groups versus ECN‐Tra + BCAAs groups (f), Ara + BCAAs groups versus ECN‐Deg + BCAAs groups (g), and Ara + CMC‐Na groups versus ECN‐Tra + BCAAs groups (h). (i)–(m) The relative richness of *Lactobacillus intestinalis* (i), *Akkermansia muciniphila* (j), *Mucispirillum schaedleri* (k), *Paramuribaculum intestinale* (l) and *Streptococcus pasteurianus* (m). CMC‐Na: sodium carboxymethyl cellulose. Statistical analysis was performed using one‐way ANOVA (*p <0.05, **p <0.01, ***p<0.001, ****p <0.0001). Data are represented as mean ± SEM.

To investigate the impact of ECN‐Deg and ECN‐Tra on the gut microbiota during treatment, we performed 16S rRNA gene sequencing analysis of the cecal contents from mice after long‐term high‐BCAAs intervention. The results indicated that ECN‐Deg and ECN‐Tra significantly altered the richness and diversity of the gut microbiota in high‐BCAAs mice (Figure 8a–c). To compare microbiota structures among the five groups, we performed ANOSIM analysis. Relative to the control without BCAAs (Ara + CMC‐Na), the ECN‐Deg + BCAAs group exhibited the most similar microbiota composition (*R* = 0.08125, *p* = 0.2546), whereas the ECN‐Con + BCAAs group showed the greatest divergence (*R* = 0.408, *p* = 0.0076). Intermediate levels of dissimilarity were observed in the Ara + BCAAs (*R* = 0.364, *p* = 0.0068) and ECN‐Tra + BCAAs (*R* = 0.184, *p* = 0.033) groups. Further analysis of the microbiota at the phylum level revealed that treatment with engineered strains reduced the richness of *Bacteroidota* (Figure 8d). At the genus level, the richness of *Ligilactobacillus* and *Paramuribaculum* in the Ara + BCAAs group was significantly lower than in the healthy group (Ara + CMC‐Na group). However, after treatment with the engineered strains, the richness of *Ligilactobacillus* and *Paramuribaculum* was significantly increased (Figure 8e).

At the species level, the ECN‐Tra treatment group showed specific enrichment of nine species compared to the Ara + BCAAs group, including *Paramuribaculum intestinale*, *Akkermansia muciniphila*, *Faecalibaculum rodentium*, and *Mucispirillum schaedleri* (Figure 8f). In the ECN‐Deg treatment group, *P. intestinale* and *F. rodentium* were specifically enriched (Figure 8g). Notably, two species, *A. muciniphila* and *F*. *rodentium*, which were significantly enriched in the ECN‐Tra treatment group, were also significantly elevated relative to the Ara + CMC‐Na group (Figure 8h). Additionally, we observed a significant increase in the richness of *Lactobacillus intestinalis* after treatment with ECN‐Deg and ECN‐Tra (Figure 8i). Following the ECN‐Tra intervention, several beneficial bacteria were enriched, such as *A. muciniphila*, *M. schaedleri*, and *P. intestinale* (Figure 8j–l). Moreover, under high‐BCAAs intervention, the harmful bacterium *Streptococcus pasteurianus* was significantly elevated, but treatment with the engineered strains (including ECN‐Con) greatly reduced its richness (Figure 8m). These results demonstrate that the engineered strains can modulate the changes in the gut microbiota structure induced by high‐BCAA intervention. Strikingly, ECN‐Tra significantly enhanced the richness of beneficial microbes. This suggests that the regulatory effect of ECN‐Tra on the gut microbiota is likely associated with the production of SCFAs.

## 3 DISCUSSION

Reducing dietary intake of BCAAs is currently the most common method used to intervene in neonatal BCAAs metabolic disorders and chronic diseases caused by excessive BCAAs. However, patient adherence to this approach is low due to the relative difficulty of restricting foods with high levels of BCAAs. With the rise of synthetic biology, live bacterial therapeutics have been increasingly developed for treating genetic metabolic diseases.

In this study, we constructed two types of engineered bacteria—ECN‐Deg and ECN‐Tra—that efficiently metabolize BCAAs using two different strategies. ECN‐Deg utilizes BCAAs as an energy substrate through de novo catabolism, whereas ECN‐Tra converts BCAAs into specific SCFAs via the *por* gene, with the resulting SCFAs providing additional intestinal benefits.^47^, ^48^ These strategies not only enhance the metabolic capacity of the chassis strain but also expand the potential applications of its metabolic products. Together, our findings demonstrate that live microbial therapeutics can effectively degrade accumulated BCAAs in the body, offering a promising alternative to dietary restriction.

When using ECN as the chassis, we knocked out the strain's native cryptic plasmid to ensure the stable inheritance of the exogenous plasmids. We then assembled various target genes into different metabolic modules, which were regulated by the Ara‐inducible promoter, enabling precise control over the expression of these metabolic modules. In our study, we knocked out genes related to the BCAAs synthesis pathway to further increase the chassis strain's demand for BCAAs. The Δ*ilvC* strain was unable to grow in the absence of BCAAs, indicating that this gene is a key factor in the BCAAs synthesis pathway in ECN. However, as ECN has multiple isoenzymes for *ilvI*, the Δ*ilvI* strain showed no significant impact in a BCAAs‐deficient environment. *lrp* activates BCAAs anabolism and inhibits BCAAs catabolism. By knocking out this gene, we comprehensively improved ECN's metabolic capacity for BCAAs. In the engineered bacterial strains of the Deg Module series, we observed that ECN neither utilizes BCAAs as preferential energy substrates nor spontaneously degrades BCAAs de novo, consistent with prior research demonstrating limited endogenous BCAAs catabolic activity in *E. coli*.^49^ Therefore, it is necessary to build a relatively complete degradation module for the engineered bacteria to utilize BCAAs. In contrast to the “Deg Module” engineered bacteria, the “Tra Module” engineered bacteria, which were constructed by introducing the *Clostridium* Por complex, metabolized BCAAs more efficiently.

In our study of BCAAs metabolism in C57BL/6 mice, we found that the levels of BCAAs are tightly regulated. Within just 2 h of large BCAAs intake, the body begins to degrade amino acids extensively, maintaining the concentration at a relatively low level. Integrating the pharmacokinetic profiles of ECN‐Deg and ECN‐Tra, we observed that the rapid systemic BCAAs clearance within 1 h post‐administration temporally correlated with the peak ileal accumulation of the engineered bacterial strains. As the last part of the small intestine, the main function of the ileum is to complete the absorption of nutrients. Therefore, by immediately administering BCAAs after bacterial dosing, we ensured that the bacteria and BCAAs encountered each other sufficiently in the ileum, facilitating the catabolism of excess BCAAs by the body. On the other hand, previous studies have indicated that both the intake of exogenous amino acids and the breakdown of endogenous proteins lead to the transport of amino acids from the blood to the gastrointestinal tract via enterohepatic circulation, which results in elevated resting amino acid levels in the intestines.^50^ In this study, we demonstrate the enterohepatic circulation of BCAAs in C57BL/6, which provides an additional opportunity for the intestine‐resident engineered bacteria to utilize BCAAs (Figure 5f–h). From the area under the curve in Figure 5c, which represents the total bacterial excretion (around 2 × 10^8^ CFU), we can deduce that a large portion of the bacteria was consumed in the stomach, with only a small fraction entering the duodenum. A similar result was found in the research of the Chen Qian team.^51^

In a study on LBPs intervention for hypertyrosinemia, Chen Peng's team confirmed the presence of intestinal circulation of Tyr in the body.^27^ In this study, we confirmed that BCAAs follow intestinal‐blood circulation. The intestinal‐blood circulation of amino acids provides a continuous supply of substrate for engineered bacteria in the gut, ensuring that engineered bacteria can efficiently metabolize the accumulated substrate. Combining together, we validated that both ECN‐Deg and ECN‐Tra had an efficient ability to utilize BCAAs in wild‐type C57BL/6 mice.

In this study, we found that elevated BCAAs increase inflammation levels in mice, while treatment with engineered bacteria reduced this inflammatory response. In a related study, Hongwei Liu's team demonstrated that oral administration of *Parabacteroides merdae* carrying the *por* gene, which efficiently degrades BCAAs, significantly reduced atherosclerotic lesions in ApoE^−/−^ mice, whereas deletion of the *por* gene abolished this protective effect.^41^ In this study, ECN‐Tra, the genetically engineered strain expressing *por*, exhibited efficiency in consuming BCAAs and alleviating inflammation‐induced damage. These findings not only demonstrate that BCAAs overload contributes to disease development and increased inflammation levels, but also reveal the therapeutic potential of engineered bacteria in addressing BCAAs‐mediated metabolic dysfunction.

Increasing evidence suggests that dysregulated gut microbiota is closely linked to colonic inflammation and intestinal barrier dysfunction.^52^ Our analysis of the gut microbiota composition revealed that the BCAA‐degrading activity of ECN‐Deg effectively counteracts the impact of BCAAs on gut microbiota, while the BCAA‐converting activity of ECN‐Tra, which produces SCFAs, significantly reshapes microbiota structure in a manner comparable to the direct effect of BCAAs. The treatment with engineered strains significantly increased the richness of *Ligilactobacillus* and *Paramuribaculum*, making the gut microbiota composition of the treated mice more similar to that of the healthy group (Ara + CMC‐Na). *Ligilactobacillus* and *Paramuribaculum* help restore the intestinal microbial balance, inhibit the growth of harmful pathogens, and protect intestinal barrier function.^53^, ^54^ Furthermore, metabolites derived from gut microbiota, such as SCFAs, have been demonstrated to provide various health benefits.^41^, ^48^, ^55^ Among these, *A. muciniphila*, which has SCFA‐producing capabilities, plays a crucial role in reducing intestinal inflammation and repairing the intestinal barrier.^56^, ^57^ *M. schaedleri* maintains gut microbial balance by competitively inhibiting the attachment and growth of pathogenic bacteria, thus protecting mice from colitis.^58^ Following ECN‐Tra intervention, the richness of *A. muciniphila* and *M. schaedleri* was significantly higher compared to other treatment groups. We hypothesize that this may be due to the SCFAs production by ECN‐Tra‐mediated BCAAs transformation, which promotes the growth of these beneficial bacteria and contributes to gut health. On the other hand, under high‐BCAAs intervention, the harmful bacterium *S. pasteurianus* showed a significant increase. This pathogen can cause various common diseases such as sepsis, meningitis, and bacteremia, and is known for its strong antibiotic resistance and pathogenicity.^59^, ^60^, ^61^, ^62^ Fortunately, intervention with engineered strains greatly reduced the richness of *S. pasteurianus*. Collectively, these results indicate that engineered bacterial intervention can change the gut microbiota composition in high‐BCAAs conditions, thereby protecting intestinal health and reducing intestinal inflammation.

## 4 LIMITATION

Our work is subject to a few limitations. The inability to directly measure intracellular BCAA levels and to determine the precise in vitro kinetics of the heterologous enzymes (BrnQ, KdcA, PrpR, Por) limits a full mechanistic understanding of the degradation flux. Additionally, the reliance on plasmid‐based expression introduces concerns regarding genetic stability and biosafety that future studies using chromosomal integration should resolve. Lastly, the consequences of the treatment‐induced microbial changes (e.g., enrichment of *A. muciniphila* and *L. intestinalis*) observed here must be interpreted with caution, as their long‐term stability and effects were not assessed in this short‐term model.

## 5 MATERIALS AND METHODS

### 5.1 Bacterial strains, plasmids, and medium

The bacterial strains involved in this study are listed in Table S1. The information on the target genes involved in the construction of plasmids is detailed in Table S2; the primers involved in the construction of plasmids are listed in Table S3. *E. coli* DH5*α* is used for the large‐scale amplification of plasmids. ECN serves as the chassis in this experiment for carrying out specific functions. M9 medium consists of 6 g/LNa_2_HPO_4_, 3 g/L KH_2_PO_4_, 0.5 g/L NaCl, 1 g/L NH_4_Cl, 5 g/L glycerol, 2 mM MgSO_4_, and 0.1 mM CaCl_2_. M9 + 30 mM BCAAs requires an additional 1.312 g/L of Leu and Ile and 0.928 g/L of Val. This medium is used to determine the consumption of BCAAs by engineered bacteria. Additionally, prepared M9 medium containing 6.8 g/L KH₂PO₄ and 10 g/L pancreatic to simulate the intestinal environment (pH 6.8) is called SIF. LB medium (5 g/L yeast extracts, 10 g/L tryptone, and 10 g/L NaCl) is used for the normal cultivation of strains. Ampicillin (Amp, 100 μg/mL), Kanamycin (Kan, 50 μg/mL), and Spectinomycin (Spec, 50 μg/mL) are all dissolved in sterile water, filtered through a 0.22 μm filter, and stored at −20°C for future use. Preparation of BCAAs solution: 0.5% CMC‐Na solution was used as the solvent. Val, Leu, and Ile were dissolved together in the 0.5% CMC‐Na solution, and sonication was performed with a temperature‐controlled ultrasonic cleaner (40°C, 400 W) until fully dissolved, resulting in a 15 mg/mL BCAAs solution.

### 5.2 ECNP genome editing and validation

We referenced the work of Yang Sheng to construct guide plasmids (pTargetFΔ*ilvC*, pTargetFΔ*ilvI*, and pTargetFΔ*lrp*) for gene editing.^63^ In the construction of the guide plasmid, we first designed the corresponding sgRNA sequences and the upstream and downstream homologous arms for different target genes and then amplified them by PCR with high‐fidelity DNA polymerase (Table S3). Subsequently, pTargetF was linearized by digestion with SpeI and HindIII and the target fragment was recombined and connected through seamless cloning. The recombined product was transformed into DH5*α*, and after selecting positive clones and extracting the plasmid, the guide plasmid pTargetFΔgene was obtained, which targets the deletion of specific genes (*ilvI*, *ilvC*, and *lrp*). The pTargetFΔ*gene* was then transformed into ECNP‐competent cells containing the pCas plasmid, and the transformed bacteria were spread onto LB plates containing 50 ng/mL Kan and 50 ng/mL Spec, and incubated at 30°C for 16 h. Finally, single clones on the dual‐antibiotic plates were selected for PCR and sequencing verification (Table S4), thereby confirming the successful gene‐edited strains.

After verifying the gene editing, the next step is to eliminate the pCas and the pTargetFΔ*gene*. The successfully edited clones are inoculated into 2 mL of LB medium containing 50 ng/mL Kan and 0.5 mM IPTG, and incubated with shaking at 30°C for 10 h. Subsequently, they are streaked onto LB plates containing Kan and incubated at 30°C for 12 h. Single clones are selected and observed for sensitivity to Spec; if sensitive to Spec, it indicates that the pTargetFΔ*gene* has been successfully eliminated. The strains containing the pCas and that have undergone one round of successful editing are preserved for subsequent secondary gene editing until the editing work is completed. The replication origin of the pCas, pCS101, is temperature‐sensitive and can be eliminated by overnight culture at 37°C.

### 5.3 Preparation and characterization of engineered strains

The constructed plasmids were transformed into strains via the heat shock method. To assess the growth curves of the engineered strains, 1% inoculum of the revived bacterial suspension was inoculated into different media containing 100 μg/mL Amp, and incubated at 37°C with shaking at 200 rpm. When the strains reached the logarithmic growth phase, 10 mM Ara was added to induce protein expression. Three biological replicates were performed for each strain. Samples were taken at specific time points, and the optical density at 600 nm (OD600) was measured using a microplate reader to monitor bacterial growth. Additionally, qPCR and WB analyses were conducted to examine the expression of the target gene.

### 5.4 In vitro cultivation of engineered bacteria in SIF

The revived bacterial strains were inoculated at 1% inoculum into SIF and incubated anaerobically at 37°C until the end of the logarithmic phase. Ara was then added to a final concentration of 10 mM, and the culture continued. At specific time points, the growth of the strains was monitored, and the BCAAs concentration in the supernatant was measured.

### 5.5 RNAseq and bioinformatics analysis

The engineered bacterial cells were cultured in SIF medium until reaching the logarithmic growth phase (8 h). The cells were then pelleted by centrifugation at 5000 × *g* for 10 min at 4°C, snap‐frozen in liquid nitrogen. The total RNA was prepared from samples using TransNGS® Ribo‐Cap rRNA Depletion Kit (Bacteria) and MagicPure® RNA Beads (TransGen Biotech, China). The library was constructed using TransNGS® Stranded RNA‐Seq Library Prep Kit for Illumina (TransGen Biotech, China). The 2 × 150 bp paired‐end sequencing was performed using the DNBSEQ‐T7.

Sequence reads from all samples were cleaned using the trimmomatic‐0.39^64^ (Phred quality score >20, reads length >50 bp). After adaptor trimming and quality trimming, the clean reads were mapped to the reference genome using Bowtie2 (version 2.3.5.1) (−‐very‐fast‐local).^65^ The DEGseq package was utilized to assess the differential gene expression between each two groups with the MARS model (MA‐plot‐based method with the random sampling model),^66^ and genes were identified as differentially expressed with at least a 2‐fold change in expression levels and FDR <0.001. Enrichment of KEGG pathways for a given gene list was calculated using a classical hypergeometric distribution statistical comparison of a query gene list against a reference gene list with a threshold of *p* <0.05.

### 5.6 Ability of BCAAs degradation for engineered bacteria in vitro

Inoculate the revived bacterial suspension into M9 medium containing 30 mM BCAAs at a 1% inoculation rate, and then continue to culture with the addition of 10 mM Ara when the OD reaches 0.4–0.6 until 24 h. During this period, samples are taken at different time points and centrifuged at 8000 × *g* for 2 min to collect the supernatant, which is used for subsequent determination of BCAAs concentrations.

### 5.7 WB assay

The bacterial strains were cultured in LB or M9 until reaching the logarithmic growth phase. Subsequently, 10 mM Ara was added to induce target gene expression. At specified time points, cells were harvested by centrifugation (5000 × *g*, 4°C, 10 min), snap‐frozen in liquid nitrogen. Then, use ultrasonication to disrupt the cells (200 W, 30 min, with a 5 s pulse and a 10 s interval). After the disruption is complete, centrifuge at 20,000 × *g* at 4°C for 10 min, and collect the supernatant, which contains the soluble proteins. For protein separation, perform SDS‐PAGE electrophoresis at 120 V for 70 min. Subsequently, transfer the separated proteins onto a PVDF membrane using electroblotting at 100 V for 70 min. Block the membrane with TBST (TBS with Tween‐20) containing 5% skim milk at room temperature for 1 h. Next, dilute the His‐tag primary antibody (66005‐1‐lg, Proteintech, 1:10,000 in TBST)/the recombinant RNA polymerase beta (RpoB) primary antibody (ab191598, Abcam, 1:1000 in TBST) and incubate with the membrane on a shaker overnight at 4°C. The following day, wash the membrane three times with TBST, each wash for 10 min, and then add the prepared secondary antibody (A0216, Beyotime, 1:1000 diluted in TBST) and incubate at room temperature for 1 h. After the incubation is complete, wash the membrane three times with TBST, each wash for 10 min. Then, incubate the membrane with ECL reagent and capture the image for analysis.

### 5.8 Quantitative real‐time PCR

The bacterial strains were cultured in LB or M9 until reaching the logarithmic growth phase. Subsequently, 10 mM Ara was added to induce target gene expression. At specified time points, cells were harvested by centrifugation (5000 × *g*, 4°C, 10 min), snap‐frozen in liquid nitrogen. Total RNA of the bacteria was extracted using TransZol, followed by cDNA synthesis using the RNA reverse transcription kit (AT341, TransGen); the reverse‐transcribed product was diluted 10‐fold with RNase‐free water and stored at −20°C. Specific primers were designed using NCBI (Table S4), and the expression of the target gene was detected by qPCR (AQ601, TransGen). Reactions were carried out on 96‐well plates with 20 μL reactions per well using a LightCycler 480 II Real‐Time PCR Detection System from Bio‐Rad (Roche, Swiss Confederation). Cycling conditions for qPCR reactions were as follows: 95°C for 30 s; 40 cycles of 95°C for 15 s, 60°C for 1 min, and 72°C for 10 s. Fluorescent measurements were taken at the end of each cycle.

### 5.9 Animal studies

The animals used in this study were purchased from Shanghai Jiesijie Laboratory Animal Co., Ltd. Male C57BL/6 mice, aged 6–7 weeks, were housed in a clean and comfortable environment (12 h light–dark cycle, temperature of 22 ± 2°C, and humidity of 40%–60%) and were allowed 1 week for acclimatization before being used in subsequent experimental procedures. The entire animal experimental process, including the purchase, breeding, and experimentation of animals, was approved by the Ethics Committee on Laboratory Animals of Shanghai Institute for Biomedical and Pharmaceutical Technologies (K2024‐81).

Investigation of BCAAs degradation by engineered strains: mice were randomly divided into four groups: (1) Ara group: mice were gavaged with 10 g/L Ara for 8 consecutive days; (2) ECN‐Con group: mice were gavaged with 2 × 10^10^ CFU of ECN‐Con for 8 consecutive days; (3) ECN‐Deg group: mice were gavaged with 2 × 10^10^ CFU of ECN‐Deg for 8 consecutive days; (4) ECN‐Tra group: mice were gavaged with 2 × 10^10^ CFU of ECN‐Tra for 8 consecutive days. On day 7, after the gavage, the mice were provided with drinking water containing 10 g/L Ara, and on day 8, they were immediately gavaged with 200 μg/g BCAAs. The volume of each administration was uniform at 200 μL per mouse. Subsequently, the mice were euthanized, and blood, feces, and ileum samples were collected for further analysis.

Investigation of the therapeutic effects of engineered strains: mice were randomly assigned to five groups: (1) Ara + CMC‐Na group: mice were daily gavaged with 10 g/L Ara and 0.5% CMC‐Na solution; (2) Ara + BCAAs group: mice were daily gavaged with 10 g/L Ara and 200 μg/g BCAAs; (3) ECN‐Con + BCAAs group: mice were daily gavaged with 2 × 10^10^ CFU of ECN‐Con and 200 μg/g BCAAs; (4) ECN‐Deg + BCAAs group: mice were daily gavaged with 2 × 10^10^ CFU of ECN‐Deg and 200 μg/g BCAAs; (5) ECN‐Tra + BCAAs group: mice were daily gavaged with 2 × 10^10^ CFU of ECN‐Tra and 200 μg/g BCAAs. The volume of each administration was uniform at 200 μL per mouse. The experiment lasted 30 days, and the engineered strains were freshly prepared each day, with interventions given at a fixed time each morning. After the intervention period, plasma, feces, liver, and ileum samples were collected for further analysis.

### 5.10 The residence of engineered strains in vivo

To investigate the metabolic behavior of engineered strains in the mouse intestine, we constructed a reporter plasmid expressing luciferase, pBAD‐*luxCDABE*. The *luxCDABE* genes were sourced from pGEN‐*luxCDABE* (Addgene: 44918) and integrated into the pBAD vector by linearizing with NheI and EcoRI, followed by homologous recombination (Table S3). The resulting reporter plasmid was transformed into ECNPΔ*ilvI*Δ*ilvC*Δ*lrp*. Subsequently, the engineered strain was orally gavaged into mice, and in vivo imaging was performed at different time points to monitor the distribution of the engineered bacteria (FUSION FX EDGE/DBT, Perkin Elmer).

The Residence of Engineered Strains: Mice were randomly divided into three groups and subjected to the following treatments: (1) Control group: Single gavage of 2 × 10^10^ CFU of ECN‐Con; (2) ECN‐Deg group: Single gavage of 2 × 10^10^ CFU of ECN‐Deg; (3) ECN‐Tra group: Single gavage of 2 × 10^10^ CFU of ECN‐Tra. Fecal samples were collected at 1, 2, 4, 8, 10, 12, 24, 36, and 48 h post‐administration. Samples were homogenized in sterile water (1 g/20,000 mL). A 100 μL aliquot of the 20,000‐fold diluted suspension was spread onto Amp plates and incubated inverted at 37°C for 24 h. Then the CFU counts were enumerated to assess bacterial clearance kinetics.

*Intestinal distribution profiling*: Mice were randomly allocated into three groups and treated as follows: (1) Control group: Single gavage of 2 × 10^10^ CFU of ECN‐Con; (2) ECN‐Deg group: Single gavage of 2 × 10^10^ CFU of ECN‐Deg; (3) ECN‐Tra group: Single gavage of 2 × 10^10^ CFU of ECN‐Tra. At 1, 3, 5, 10, 12, and 24 h post‐treatment, three mice per group were sacrificed. Luminal contents from the jejunoileal segment (jejunum + ileum), cecum, and colon were collected, homogenized in sterile water diluted 20,000‐fold. A 100 μL aliquot of the diluted suspension was plated on Amp plates and incubated inverted at 37°C for 24 h. Then the CFU counts were performed to quantify bacterial load in distinct intestinal regions.

### 5.11 Verification of BCAAs intestinal‐blood circulation

Isotopically labeled ^13^C‐BCAAs (^13^C‐Val, ^13^C‐Leu and ^13^C‐Ile) were intravenously injected into C57BL/6 mice (20 μg/g of ^13^C‐BCAAs). At designated time points, the mice were euthanized, and blood and small intestine effluents were collected. Whole blood was collected in anticoagulant tubes, and plasma was separated by centrifugation at 15,000 × *g* for 10 min at 4°C. Finally, the concentrations of ^13^C‐BCAAs in the intestinal effluents and plasma were quantitatively analyzed.

### 5.12 Detection of ALT and AST in the liver

Liver tissue (0.1 g) was collected from the same location, washed with PBS to remove any debris, and stored at −80°C for future use. The levels of alanine aminotransferase (ALT) (BC1555, solarbio) and aspartate aminotransferase (AST) (BC1565, solarbio) in the liver were then measured according to the instructions provided by the respective assay kits.

### 5.13 Histology of the ileum

After euthanizing the mice, the ileum was immediately collected and placed in tissue fixative, and fixed at 4°C for 24 h. The samples were then embedded and sectioned, followed by hematoxylin and eosin (H&E) staining. Finally, the tissue structure was observed under a light microscope.

### 5.14 Luminex liquid suspension chip detection

Luminex liquid suspension chip detection was performed by Wayen Biotechnologies (Shanghai, China) using the Bio‐Plex Pro Mouse Chemokine Panel 23‐plex kit, following the manufacturer's instructions. Briefly, samples were added to a 96‐well plate and incubated with beads for 1 h, followed by incubation with detection antibodies for 30 min. Streptavidin‐PE was then added to each well and incubated for 10 min. Finally, the data were read using the Bio‐Plex MAGPIX system.

### 5.15 BCAAs quantification

BCAAs quantification was performed by mass spectrometry, and the measurement represents the combined levels of isoleucine, valine, and leucine. An appropriate volume of the mixed sample (0.1 mL) was added to 0.9 mL of 50% acetonitrile, ground for 5 min, vortex‐mixed, and centrifuged at 10,000 × *g* for 10 min. The supernatant was passed through a 0.22 μm micropore filter, followed by analysis using a Waters BEH Amide column (2.1 mm × 100 mm × 1.7 μm). The column temperature was set to 35°C, and the flow rate was 0.35 mL/min. Electrospray ionization (ESI+) mode was used for detection. The concentration of BCAAs was calculated using the following formula:

$$W=\frac{C*V*N}{m*1000}$$

*W* is the concentration of the target compound in the sample (mg/L); *C* is the concentration of the target compound in the test solution (mg/L); *V* is the final volume (mL); *N* is dilution factor; *m* is mass of the sample (mL).

### 5.16 16S rRNA gene sequencing analysis of gut microbiota

Mice were randomly divided into five groups with 30 days of BCAAs intervention, fecal samples were collected from the mice, and stored at −80°C before DNA extraction. Genomic DNA was extracted from 30 mg of feces using a QIAamp DNA Stool Mini Kit (Qiagen, Hilden, Germany). The primers 338F (“ACTCCTACGGGAGGCAGCAG”) and 806R (“GGACTACHVGGGTWTCTAAT”) were used to amplify the hypervariable V3‐V4 region of the 16S rRNA gene. Sequencing was performed on an Illumina NextSeq2000 instrument with 2 × 300 cycles.

To analyze the microbiota data, raw paired FASTQ files were treated using QIIME 2, and amplicon sequence variants (ASVs) were generated using the DADA2 plugin with default parameters.^67^ The minimum sample size was the criterion for data normalization. Community richness, evenness, and diversity analyses (Shannon, Simpsonenven, ACE, Chao and Good's coverage) were performed using Mothur (version 1.48.2).^68^ Taxonomy was assigned using the RDP classifier (version 2.14, August 2023)^69^ with the default parameter (80% threshold) based on the Ribosomal Database Project.^70^ The species was identified using BLASTN with the SILVA database (version 138.2) and NCBI, and the best hit was chosen with an identity score >97% and an alignment score >97%. Permutational multivariate analysis of variance (PERMANOVA) was used to determine differences between microbial communities based on the Bray–Curtis distance matrix, which was subsequently analyzed by principal coordinate analysis. The differences in taxonomic abundance between groups were analyzed using LEfSe (http://huttenhower.sph.harvard.edu/galaxy/).^71^

### 5.17 Statistics analysis

Using SPSS for significance analysis, the differences between groups were compared using One‐way ANOVA, followed by multiple comparisons of each group sample using SNK. A *p*‐value of <0.05 was considered statistically significant. Using GraphPad Prism 9 and Adobe Illustrator 2023 for data visualization.

## AUTHOR CONTRIBUTIONS

**Zhaowei Chen:** Conceptualization, Methodology, Data curation, Investigation, Visualization, Writing‐original draft. **Jingyi Xu:** Investigation, Data curation. **Huayue Zhang**: Validation, Data Curation, **Yuezhu Wang:** Formal Analysis, Visualization. **Mingjie Li:** Investigation. **Yixiao Wu:** Investigation. **Yongqiang Zhu**: Investigation. **Yue Liu:** Validation. **Haiyang Xi**a: Methodology, Writing—Review and Editing, Project Administration. **Huajun Zheng**: Conceptualization, Methodology, Resources, Writing—Review and Editing, Supervision, Funding Acquisition.

## CONFLICT OF INTEREST STATEMENT

The authors state that none of the work presented in this study may have been influenced by any known conflicting financial interests or personal relationships.

## Supporting information


**Figure. S1.** Construction of pTargetFΔ*gene* guide plasmids and PCR validation. (A) Schematic of guide plasmid construction; (B), (C) PCR and sequencing validation of Δ*ilvC* clones; (D), (E) PCR and sequencing validation of Δ*ilvI* clones; (F), (G) PCR and sequencing validation of Δ*lrp* clones. M: Trans 2K Plus II DNA marker; P: Positive clone; N: Negative clone.
**Table S1.** Strains and plasmids involved in this study.
**Table S2.** Information on target genes involved in pathway construction.
**Table S3.** Information on primers involved in plasmid construction.
**Table S4.** Primers for gene editing verification.
**Table S5.** Primers for qPCR.

## Data Availability

The raw data of RNAseq and 16S rRNA gene sequencing have been submitted to the GenBank Sequence Read Archive (accession number PRJNA1216379).
