# Supplementary material for: Design and pharmacodynamic study of live biotherapeutic products with efficient degradation of branched‐chain amino acids
Source: Bioeng Transl Med. 2025 Sep 15;10(6):e70075. doi: 10.1002/btm2.70075 (PMC12617562; doi:10.1002/btm2.70075)
Supplement: Supplementary file 1 — Figure. S1. Construction of pTargetFΔgene guide plasmids and PCR validation. (A) Schematic of guide plasmid construction; (B), (C) PCR and sequencing validation of ΔilvC clones; (D), (E) PCR and sequencing validation of ΔilvI clones; (F), (G) PCR and sequencing validation of Δlrp clones. M: Trans 2K Plus II DNA marker; P: Positive clone; N: Negative clone. Table S1. Strains and plasmids involved in this study. Table S2. Information on target genes involved in pathway construction. Table S3. Information on primers involved in plasmid construction. Table S4. Primers for gene editing verification. Table S5. Primers for qPCR. [file BTM2-10-e70075-s001.docx]

**Design and pharmacodynamic study of live biotherapeutic products with efficient degradation of branched-chain amino acids**

Zhao-Wei Chen^1^, Jing-Yi Xu^1^, Hua-Yue Zhang^1^, Yue-Zhu Wang^1^, Ming-Jie Li^1^, Yi-Xiao Wu^1^, Yong-Qiang Zhu^1^, Yue Liu^1^, Hai-Yang Xia^1*^, Hua-Jun Zheng^1*^

^1^Shanghai-MOST Key Laboratory of Health and Disease Genomics, NHC Key Lab of Reproduction Regulation, Shanghai Institute for Biomedical and Pharmaceutical Technologies, School of Basic Medical Sciences, Fudan University, Shanghai, 200032, China

*Corresponding author’s email: Hai-Yang Xia: [hyxia@sibpt.com](mailto:hyxia@sibpt.com), Hua-Jun Zheng: [zhenghj@chgc.sh.cn](mailto:zhenghj@chgc.sh.cn).

**Supplementary information**


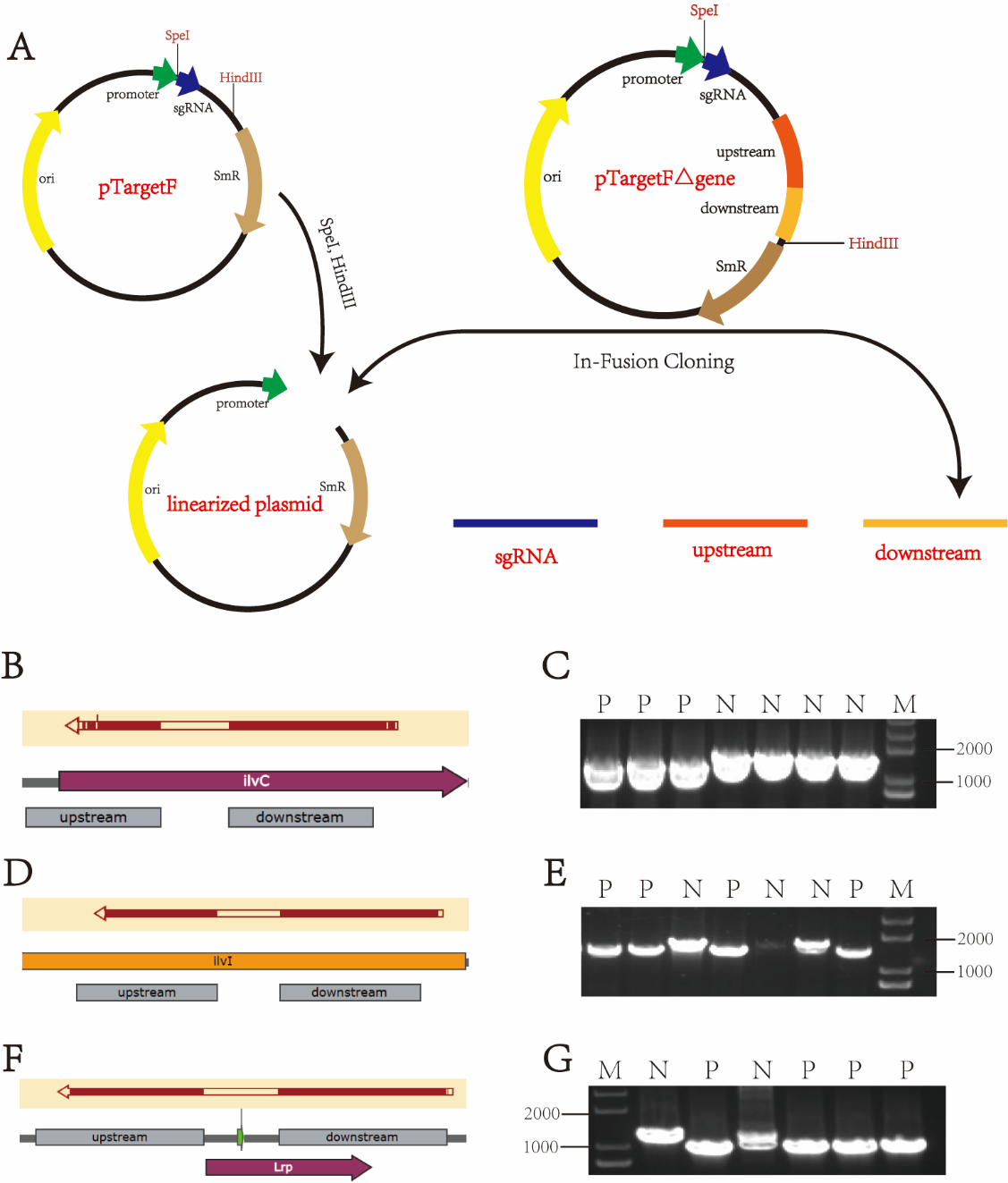


**Fig. S1. Construction of pTargetFΔ*gene* guide plasmids and PCR validation.** (A): Schematic of guide plasmid construction; (B-C): PCR and sequencing validation of Δ*ilvC* clones; (D-E): PCR and sequencing validation of Δ*ilvI* clones; (F-G): PCR and sequencing validation of Δ*lrp* clones. M: Trans 2K Plus II DNA marker; P: Positive clone; N: Negative clone.

**Table S1.** Strains and plasmids involved in this study**.**

| **Plasmid or Strain** | **Abbreviations** | **Character** | **Source** |
| --- | --- | --- | --- |
| **Plasmid** |  |  |  |
| pBAD | — | *AmpR*,araC,ColE1/pMB1/pBR322/pUC origin | Lab stock |
| pBAD- *kdcA* | pBAD-K | *AmpR*, *kdcA* expression under ParaBAD/araC | This study |
| pBAD- *brnQ* | pBAD-B | *AmpR*, *brnQ* expression under ParaBAD/araC | This study |
| pBAD- *prpR* | pBAD-P | *AmpR*, *prpR* expression under ParaBAD/araC | This study |
| pBAD- *kdcA* - *brnQ* | pBAD-KB | *AmpR*, *kdcA* and *brnQ* expression under ParaBAD/araC | This study |
| pBAD- *kdcA* - *brnQ* - *prpR* | pBAD-KBP | *AmpR*, *kdcA*, *brnQ* and *prpR* expression under ParaBAD/araC | This study |
| pBAD- *brnQ* - *por* | pBAD-BpA | *AmpR*, *brnQ*, *fes* and *por* expression under ParaBAD/araC | This study |
| pBAD-*LuxCDABE* | pBAD-Lux | *AmpR*, *LuxCDABE* expression under ParaBAD/araC | This study |
| pCas | — | repA101(Ts) kan Pcas-cas9 ParaB-Red lacIq Ptrc-sgRNA-pMB1 | Gift from Yang Sheng Lab |
| pTargetF | — | *SpecR*, gRNA template plasmid for N20 modification | Gift from Yang Sheng Lab |
| pTargetT-Δ*lrp* | — | pMB1 aadA sgRNA-*lrp* Δ*lrp* (1000 bp) | This study |
| pTargetT-Δ*ilvI* | — | pMB1 aadA sgRNA-*ilvI* Δ*ilvI* (1000 bp) | This study |
| pTargetT-Δ*ilvC* | — | pMB1 aadA sgRNA-*ilvC* Δ*ilvC* (1013 bp) | This study |
| **Strain** |  |  |  |
| *E. coli DH5α* | — | F- *endA1 glnV44 thi-1 recA1 relA1 gyrA96 deoR nupG Φ80dlacZ* ΔM15Δ(*lacZYA-argF*)*U169 hsdR17* (rK- mK+) λ- | Lab stock |
| *E. coli Nissle* 1917 (ECN) | ECN | *E. coli* O6:K5:H1 | Lab stock |
| *Lactococcuslactissubsp.Cremoris* | — | wild type | Lab stock |
| *Clostridium sporogenes* | *—* | *wild type* | Lab stock |
| ECNΔpMUT1ΔpMUT2 | ECNP | EcN 1917 without pMUT1 and pMUT2 plasmid | Gift from FengQing Wang Lab |
| ECNPΔ*ilvI* | — | ECNP with *ilvI* deletion | This study |
| ECNPΔ*ilvC* | — | ECNP with *ilvC* deletion | This study |
| ECNPΔ*lrp* | — | ECNP with *lrp* deletion | This study |
| ECNPΔ*ilvI*Δ*ilvC* | — | ECNP with *ilvI, ilvC* deletion | This study |
| ECNPΔ*ilvI*Δ*lrp* | — | ECNP with *ilvI, lrp* deletion | This study |
| ECNPΔ*ilvC*Δ*lrp* | — | ECNP with *ilvIC, lrp* deletion | This study |
| ECNPΔ*ilvI*Δ*ilvC*Δ*lrp* | — | ECNP with *ilvI, lvIC, lrp* deletion | This study |

**Table S2.** Information on target genes involved in pathway construction.

| **Gene name**: *brnQ*;  **Source**: *E. coli Nissle 1917*;  **Sequence ID or Location**: GCF_000714595.1 and locus_tag=ECOLIN_RS02420;  **Gene sequence**:  ATGACCCATCAATTAAGATCGCGCGATATCATCGCTCTGGGCTTTATGACATTTGCGTTGTTCGTCGGCGCAGGTAACATTATTTTCCCTCCAATGGTCGGCTTGCAGGCAGGCGAACACGTCTGGACTGCGGCATTCGGCTTCCTCATTACTGCCGTTGGCCTACCGGTATTAACGGTAGTGGCGCTGGCAAAAGTTGGCGGCGGTGTTGACAGTCTCAGCACGCCAATTGGTAAAGTCGCTGGCGTACTGCTGGCAACAGTTTGTTACCTGGCGGTGGGGCCGCTTTTTGCTACGCCGCGTACAGCTACCGTTTCTTTTGAAGTGGGCATTGCGCCGCTGACGGGTGATTCCGCGCTGCCGCTGTTTATTTACAGCCTGGTCTATTTCGCTATCGTTATTCTGGTTTCGCTCTATCCGGGCAAGCTGCTGGATACCGTGGGCAACTTCCTTGCGCCGCTGAAAATTATCGCGCTGGTCATCCTGTCTGTTGCCGCAATTATCTGGCCGGCGGGTTCTATCAGTACGGCGACTGAGGCTTATCAAAACGCTGCGTTTTCTAACGGCTTCGTCAACGGCTATCTGACCATGGATACGCTGGGCGCAATGGTGTTTGGTATCGTTATTGTTAACGCGGCGCGTTCTCGTGGCGTTACCGAAGCGCGTCTGCTGACCCGTTATACCGTCTGGGCTGGCCTGATGGCGGGTGTTGGTCTGACTCTGCTGTACCTGGCGCTGTTCCGTCTGGGTTCAGACAGCGCGTCGCTGGTCGATCAGTCTGCAAACGGTGCGGCGATCCTGCATGCTTACGTTCAGCATACCTTTGGCGGCGGCGGTAGCTTCCTGCTGGCGGCGTTAATCTTCATCGCCTGCCTGGTCACGGCGGTTGGCCTGACCTGTGCTTGTGCAGAATTCTTCGCCCAGTACGTACCGCTCTCTTATCGTACGCTGGTGTTTATCCTCGGCGGCTTCTCGATGGTGGTGTCTAACCTCGGCTTGAGCCAGCTGATTCAGATCTCTGTACCGGTGCTGACCGCCATTTATCCGCCGTGTATCGCACTGGTTGTATTAAGTTTTACACGCTCATGGTGGCATAATTCGTCCCGCGTGATTGCTCCGCCGATGTTTATCAGCCTGCTTTTTGGTATTCTCGACGGGATCAAGGCATCTGCATTCAGCGATATCTTACCGTCCTGGGCGCAGCGTTTACCGCTGGCCGAACAAGGTCTGGCGTGGTTAATGCCAACAGTGGTGATGGTGGTTCTGGCCATTATCTGGGATCGTGCGGCAGGTCGTCAGGTGACCTCCAGCGCTCACTAA  **Protein sequence**:  MTHQLRSRDIIALGFMTFALFVGAGNIIFPPMVGLQAGEHVWTAAFGFLITAVGLPVLTVVALAKVGGGVDSLSTPIGKVAGVLLATVCYLAVGPLFATPRTATVSFEVGIAPLTGDSALPLFIYSLVYFAIVILVSLYPGKLLDTVGNFLAPLKIIALVILSVAAIIWPAGSISTATEAYQNAAFSNGFVNGYLTMDTLGAMVFGIVIVNAARSRGVTEARLLTRYTVWAGLMAGVGLTLLYLALFRLGSDSASLVDQSANGAAILHAYVQHTFGGGGSFLLAALIFIACLVTAVGLTCACAEFFAQYVPLSYRTLVFILGGFSMVVSNLGLSQLIQISVPVLTAIYPPCIALVVLSFTRSWWHNSSRVIAPPMFISLLFGILDGIKASAFSDILPSWAQRLPLAEQGLAWLMPTVVMVVLAIIWDRAAGRQVTSSAH* |
| --- |
| **Gene name**: *prpR*;  **Source**: *E. coli Nissle 1917*;  **Sequence ID or Location**: GCF_000714595.1 and locus_tag=ECOLIN_RS02145;  **Gene sequence**:  ATGGCACATCCACCACGGCTGAATGACGACAAACCGGTTATCTGGACGGTATCTGTAACGCGGCTGTTCGAGCTGTTTCGCGATATCAGCCTCGAGTTTGATCATCTGGCGAACATCACCCCTATTCAGCTTGGCTTTGAAAAAGCGGTGGCCTACATCCGCAAAAAACTGGCCAGCGAACGCTGCGACGCCATCATTGCCGCTGGCTCTAACGGTGCGTACCTGAAAAGCCGCCTGTCGGTACCGGTGATTCTGATAAAACCGAGTGGCTACGATGTGTTACAGGCGCTGGCAAAAGCCGGAAAACTCACCTCTTCTATCGGCGTTGTCACCTATCAGGAAACCATTCCGGCACTGGTAGCGTTTCAAAAAACCTTTAATTTGCGCCTCGACCAACGTAGCTACATTACCGAAGAAGACGCACGCGGGCAGATTAACGAGCTAAAAGCCAACGGCACCGAAGCGGTGGTCGGCGCGGGGCTGATTACCGATCTGGCAGAAGAAGCCGGAATGACCGGAATTTTTATCTATTCTGCCGCCACCGTGCGCCAGGCATTTAGCGATGCGCTGGATATGACGCGCATGTCGTTACGCCATAACACTCACGATGCCACCCGCAACGCCCTGCGTACTCGTTACGTGCTGGGCGATATGCTCGGTCAATCACCACAGATGGAACAGGTGCGGCAGACTATTTTGCTGTATGCCCGCTCCAGTGCGGCGGTGTTGATTGAGGGGGAAACGGGGACGGGCAAAGAGCTGGCGGCCCAGGCGATTCATCGAGAATATTTTGCCCGCCACGATGTGCGACAGGGCAAAAAGTCACATCCGTTTGTTGCCGTCAACTGCGGGGCGATTGCCGAATCGCTGCTGGAAGCAGAGCTGTTTGGCTATGAGGAAGGGGCGTTCACCGGCTCGCGACGCGGAGGTCGCGCCGGGCTGTTCGAAATTGCCCACGGCGGTACGCTGTTTCTGGATGAGATTGGCGAAATGCCGCTGCCGTTGCAGACTCGCCTGTTACGGGTGCTGGAAGAAAAAGAGGTCACCCGCGTCGGCGGGCATCAGCCTGTTCCGGTAGATGTGCGGGTCATTAGCGCCACTCACTGCAATCTGGAAGAAGATATGCAGCAAGGGCAGTTTCGCCGTGACCTGTTTTATCGGCTGAGTATTTTACGCCTGCAACTGCCGCCGCTACGCGAGCGGGTGGCGGATATTCTGCCGCTGGCGGAAAGCTTTTTGAAAATGTCTCTGGCGGCACTCTCTGTCCCTTTTTCTGCCGCATTACGTCAGGGATTAGAGACCTGTCAGATTGTATTATTGCTCTACGACTGGCCGGGCAATATTCGTGAACTGCGCAATATGATGGAACGACTGGCGTTATTTTTAAGTGTGGAACCGACGCCGGATTTAACGCCGCAATTTTTGCAGCTGCTACTGCCGGAACTGGCGCGCGAGTCGGCGAAGACTCCCATTCCAGGCTTGCTGACAGCACAACAGGCACTGGAGAAATTTAATGGCGATAAGACAGCAGCGGCGAATTATTTAGGTATCAGCCGGACGACGTTCTGGCGGCGGCTGAAAAGCTGA  **Protein sequence**:  MAHPPRLNDDKPVIWTVSVTRLFELFRDISLEFDHLANITPIQLGFEKAVAYIRKKLASERCDAIIAAGSNGAYLKSRLSVPVILIKPSGYDVLQALAKAGKLTSSIGVVTYQETIPALVAFQKTFNLRLDQRSYITEEDARGQINELKANGTEAVVGAGLITDLAEEAGMTGIFIYSAATVRQAFSDALDMTRMSLRHNTHDATRNALRTRYVLGDMLGQSPQMEQVRQTILLYARSSAAVLIEGETGTGKELAAQAIHREYFARHDVRQGKKSHPFVAVNCGAIAESLLEAELFGYEEGAFTGSRRGGRAGLFEIAHGGTLFLDEIGEMPLPLQTRLLRVLEEKEVTRVGGHQPVPVDVRVISATHCNLEEDMQQGQFRRDLFYRLSILRLQLPPLRERVADILPLAESFLKMSLAALSVPFSAALRQGLETCQIVLLLYDWPGNIRELRNMMERLALFLSVEPTPDLTPQFLQLLLPELARESAKTPIPGLLTAQQALEKFNGDKTAAANYLGISRTTFWRRLKS* |
| **Gene name:** *kcdA;*  **Source:** *Lactococcus lactis subsp. Lactis*;  **Sequence ID or Location:** AJ746364.1;  **Gene sequence:**  ATGTATACAGTAGGAGATTACCTATTAGACCGATTATACGAGTTAGGAATTGAAGAAATATTTGGAGTCCCTGGAGACTATAACTTACAATTTTTAGATCAAATTATTTCCCGCAAGGATATGAAATGGGTCGGAAATGCTAATGAATTAAATGCTTCATATATGGCTGATGGCTATGCTCGTACTAAAAAAGCTGCCGCATTTCTTACAACCTTTGGAGTAGGTGAATTGAGTGCAGTTAATGGATTAGCAGGAAGTTACGCCGAAAATTTACCAGTAGTAGAAATAGTGGGATCACCTACATCAAAAGTTCAAAATGAAGGAAAATTTGTTCATCATACGCTGGCTGACGGTGATTTTAAACACTTTATGAAAATGCACGAACCTGTTACAGCAGCTCGAACTTTACTGACAGCAGAAAATGCAACCGTTGAAATTGACCGAGTACTTTCCGTACTACTAAAAGAAAGAAAACCTGTCTATATCAACTTACCAGTTGATGTTGCTGCTGCAAAAGCAGAGAAACCCTCACTCCCTTTGAAAAAAGAAAATCCAAATTCAAATACAAGTGACCAAGAGATTTTGAACAAAATTCAAGAAAGCTTGAAAAATGCCAAAAAACCAATCGTGATTACAGGACATGAAATAATTAGTTTTGGCTTAGAAAAAACAGTCACTCAATTTATTTCAAAGACAAAACTACCTATTACGACATTAAACTTTGGTAAAAGTTCAGTTGATGAAGCTCTCCCTTCATTTTTAGGAATCTATAATGGTAAACTCTCAGAGCCTAATCTTAAAGAATTCGTGGAATCAGCCGACTTCATCCTGATGCTTGGAGTTAAACTCACAGACTCTTCAACAGGAGCCTTCACTCATCATTTAAATGAAAATAAAATGATTTCACTGAATATAGATGAAGGAAAAATATTTAATGAAAGAATCCAAAATTTTGATTTTGAATCCCTCATCTCCTCTCTCTTAGACCTAAGCGAAATAGAATACAAGGGAGAATATATCAATAAAAAGCAAGAAAACTTTGTTCCATCAAATGCCCTTTTATCACAAGACCGCCTATGGCAAGCAGTTGAAAATTTAACTCAAAGTAATGAAACAATCGTTGCTGAACAAGGAACATCATTCTTTGGCGCTTCATCAATTTTCTTAAAACCAAAGAGTCATTTTATTGGTCAACCCTTATGGGGATCAATTGGATATACTTTCCCAGCAGCATTAGGAAGCCAAATTGCAGATAAAGAAAGCAGACACCTTTTATTTATTGGTGACGGTTCACTTCAACTTACGGTGCAAGAATTAGGATTAGCAATCAGAGAAAAAATTAATCCAATTTGTTTTATTATCAATAATGATGGTTATACAGTTGAAAGAGAAATTCATGGACCAAATCAAAGCTACAATGATATTCCAATGTGGAATTACTCAAAATTACCAGAATCATTTGGAGCAACAGAAGATCGAGTAGTCTCAAAAATCGTTAGAACTGAAAATGAATTTGTGTCTGTCATGAAAGAAGCTCAAGCAGATCCAAATAGAATGTATTGGATTGAGTTAGTTTTGGCAAAAGAAGATGCACCAAAAGTACTGAAAAAAATGGGTAAACTATTTGCTGAACAAAATAAATCACATTGA  **Protein sequence:**  MYTVGDYLLDRLYELGIEEIFGVPGDYNLQFLDQIISRKDMKWVGNANELNASYMADGYARTKKAAAFLTTFGVGELSAVNGLAGSYAENLPVVEIVGSPTSKVQNEGKFVHHTLADGDFKHFMKMHEPVTAARTLLTAENATVEIDRVLSVLLKERKPVYINLPVDVAAAKAEKPSLPLKKENPNSNTSDQEILNKIQESLKNAKKPIVITGHEIISFGLEKTVTQFISKTKLPITTLNFGKSSVDEALPSFLGIYNGKLSEPNLKEFVESADFILMLGVKLTDSSTGAFTHHLNENKMISLNIDEGKIFNERIQNFDFESLISSLLDLSEIEYKGEYINKKQENFVPSNALLSQDRLWQAVENLTQSNETIVAEQGTSFFGASSIFLKPKSHFIGQPLWGSIGYTFPAALGSQIADKESRHLLFIGDGSLQLTVQELGLAIREKINPICFIINNDGYTVEREIHGPNQSYNDIPMWNYSKLPESFGATEDRVVSKIVRTENEFVSVMKEAQADPNRMYWIELVLAKEDAPKVLKKMGKLFAEQNKSH* |
| **Gene name:** *por;*  **Source:** *Clostridium sporogenes* ATCC 15579;  **Sequence ID or Location:** GCF_000155085.1 and locus_tag=CLOSPO_00147~ CLOSPO_00149;  **Gene sequence:**  ATGGGTGAAAAAGTTTTAATGAAGGGTAATGAAGCTATAGGCGAAGCTGCAATCCAAGCAGGATGTGAATGTTTCTTTGGGTATCCAATTACTCCACAAACAGAAGTAGCAGCTTATATGTCAAAGAAAATGCCTAAGATAGGCAAGACATTTGTTCAAGCAGAAAGTGAAATATCAGCTGTAAATATGGTGTATGGTGCAGCAGGAACAGGAATTAGATGTATGACTTCCTCAAGTTCACCAGGAATAAGTTTAAAATCAGAAGGACTTTCATATATAGCAGCAGCAGAACTACCATGTGTTATAATAAACATCGTTAGAGGAGGTCCAGGATTAGGAAGTATTCAGCCAGCACAATCAGATTATTTCCAAGCAACAAAAGCAAGCGGACACGGTGATTTTAATATGCCAGTATTTGCACCTGCTTCTATACAAGAAATGGTTGATTTAATACAAAATGCCTTTGATGTAGCTGATACATACAGGACACCTTGTATGGTTATGGGAGATGGTATGCTTGGACAAATGATGGAACCTGTTGAATTTAAAGAAAGATCATCTAAAGAACTTCCAGCAAAAGATTGGGCAGCTAATGGGTTACATGGAAGAAAAGAGCATAATATAATAAACTCCTTATATTTACAACCAGAAATATTAGAACAACATAACATTCATTTACAAGATAAATATGCTAAGATAAAAGAAAATGAAGTTAGATATGAGTTATATAACTGTGATAAAGAATGCGATTTAATATTAGTTGCATATGGAACAACTTCAAGAATATGCAAAAATGTTGTAAAAATGGCTAAAGAAGAAGGTATGACATTAGGACTAATAAGACCTATAACAATATGGCCTTTCCCATTTGAAGCATTTGAAAAAACTGTAGACCTTACTAAACATGGATATCTGTCTGTAGAAATGAGTTGCGGACAAATGGTTTATGATGTTAAATTAGCATCTAATGGAAGAAAGCCAGTAGATTTTTATGGAAGAACAGGTGGAATGGTTCCAGATCCATCAGATATCCTAGAGAAAGTTAAATCTATAGTAGGAGGTGCTAGATAATGGCTATAGTATATCAACCACCTAAAGCATTAATGGACGTTCCTACACATTATTGTCCAGGATGTACTCATGGTGTAATTCATAAATTAGTTGGAGAAGTAATTGATGAACTTGGAGTATTAGATAAAACAATAGGTGTTGCTCCAGTTGGATGTTCAGTTTTAGCATATAACTATTTTGCCTGTGATATGTTTGAGGCTGCTCATGGTAGAGCACCAGCAGTTGCAACAGGTATAAAAAGAGCTAATCCAGATTCAGTAGTATTTACTTATCAAGGAGATGGAGACTTAGCTGCAATAGGTACAGCTGAAATAGTTCACATTGCAACTAGAGGAGAAAATATTACAACAATATTCGTAAATAACTGTATATATGGAATGACAGGTGGACAGATGGCACCTACTACATTACCAGGTCAAGTAACAGAAACAACACCTTATGGAAGAGATACAAGTTATGCAGGATTCCCAATAAGAGTAGCAGAAATGATATCTACTCTAACTGGAGCTTGCTATGTAGAAAGAGTAGCTGTAAATACAGTTCCAAATATATTAAAAGCTAAAAAGGCAATAAAGAAAGCTTTCCAAAATCAAATAGATAAAAAAGGATTTTCTTTAGTTGAAGTATTGTCAATATGCCCAACTAACTGGGGATTAACTCCTCAAGAATCAATGGACTGGTTAAGAGAAAACATGATCCCATACTATCCTCTTGGCGTTAAGAAGGATACAACTGAGGAGGTGAAATAATATGGCATCACAACAAATTATATTTGCAGGTTTTGGAGGCCAAGGTATATTATCAATGGGTAAATTTTTAGCTTATGCAGGAATGGACTCAAATATGGAAGTTTCATGGTTACCATCTTACGGACCAGAAATGAGAGGTGGTACAGCTAACTGTTCTGTAGTTCTATCTGATACACCAGTTGGATCACCAATAGTAACTAAACCAGATACAGTAGTAGTAATGAATAGACCTTCTTTAGATAAATTTGAAGATATGGTAGCACCAGGTGGATTAATAATATTGGATTCTGACTTAGTTGATAGAATGCCAAAAAGAGATGACATAAAAGTTATAGCTATACCAGCTCAATCTGAAGCGGATAAAATAAGTAGTAAAAAAATAGCTAATATGATTCTTTTGGGAGCTCTTGTAAAACAAACAGGAATAGTTACTATGGATGAAATAACAGCATCATTAAAAGACCATGGTAAGGAAAAATTCTTTGAATTAAACAAAGAAGCTCTTAAAGCTGGAGAGGAATACGTAAAA  **Protein sequence:**  MGEKVLMKGNEAIGEAAIQAGCECFFGYPITPQTEVAAYMSKKMPKIGKTFVQAESEISAVNMVYGAAGTGIRCMTSSSSPGISLKSEGLSYIAAAELPCVIINIVRGGPGLGSIQPAQSDYFQATKASGHGDFNMPVFAPASIQEMVDLIQNAFDVADTYRTPCMVMGDGMLGQMMEPVEFKERSSKELPAKDWAANGLHGRKEHNIINSLYLQPEILEQHNIHLQDKYAKIKENEVRYELYNCDKECDLILVAYGTTSRICKNVVKMAKEEGMTLGLIRPITIWPFPFEAFEKTVDLTKHGYLSVEMSCGQMVYDVKLASNGRKPVDFYGRTGGMVPDPSDILEKVKSIVGGAR*MAIVYQPPKALMDVPTHYCPGCTHGVIHKLVGEVIDELGVLDKTIGVAPVGCSVLAYNYFACDMFEAAHGRAPAVATGIKRANPDSVVFTYQGDGDLAAIGTAEIVHIATRGENITTIFVNNCIYGMTGGQMAPTTLPGQVTETTPYGRDTSYAGFPIRVAEMISTLTGACYVERVAVNTVPNILKAKKAIKKAFQNQIDKKGFSLVEVLSICPTNWGLTPQESMDWLRENMIPYYPLGVKKDTTEEVK*MASQQIIFAGFGGQGILSMGKFLAYAGMDSNMEVSWLPSYGPEMRGGTANCSVVLSDTPVGSPIVTKPDTVVVMNRPSLDKFEDMVAPGGLIILDSDLVDRMPKRDDIKVIAIPAQSEADKISSKKIANMILLGALVKQTGIVTMDEITASLKDHGKEKFFELNKEALKAGEEYVK* |
| **Gene name:** *feS;*  **Source:** *Clostridium sporogenes* ATCC 15579;  **Sequence ID or Location:** GCF_000155085.1 and locus_tag=CLOSPO_00146;  **Gene sequence:**  ATGCTCTGTTTTGGAGTGATTAAAATGAGTAGAATAAATATCTTTACAGGACATTTTGGTAGTGGAAAAACAGAAATTGCCATAAATTATGCTATGAAGTTAGCGAAAGAAGGCAAAAAGGTAGCTTTAGTAGACATAGATATTGTAAATCCATATTTCTGCTCAAGAAGTCTAAAGGAAGAGTTTGATAAATTAGGAATAAGAGTAATAGCTTCTGACTCTAAACTTATGAATGCAGAATTAATGGTTGTACCTGGTGAAGTTATGGCAGTATTCAATGATAAAAGTTATGAGGTTGTTATGGATATAGGTGGTGATGATCAAGGAGCAACAGTACTTGGTCAATATAACAAATATTTTAATGAAGAAGATTATGATATGTATTTTGTAGTTAATAATAATAGACCACTTACATCTAATGAAAAAGAAACAGAAGATTATATAAAATCTATAGAAATTTCATCTAGATTAAAAGTTAAATATCTTATATCAAATACAAATCTTTCATATGAAACCACAGTAGATCACATATTAAAGGGTGATGAGATAGTTTTAGAACTTTCTAAAAAGACTGGTCTTCCCTATAAATATATTGTGTGTAGAAAAGATTTTCTAGATGATATAAAAGGTAAGGTTCATGGAGAAATATTTCCAATAGATATATACATGAAACCACCTTGGAGGCATTAA  **Protein sequence:**  MLCFGVIKMSRINIFTGHFGSGKTEIAINYAMKLAKEGKKVALVDIDIVNPYFCSRSLKEEFDKLGIRVIASDSKLMNAELMVVPGEVMAVFNDKSYEVVMDIGGDDQGATVLGQYNKYFNEEDYDMYFVVNNNRPLTSNEKETEDYIKSIEISSRLKVKYLISNTNLSYETTVDHILKGDEIVLELSKKTGLPYKYIVCRKDFLDDIKGKVHGEIFPIDIYMKPPWR* |

**Table S3.** Information on primers involved in plasmid construction.

| prime name | sequence 5'-3' | function |
| --- | --- | --- |
| K1 | ATCATCATCATCATGGTATGGCTAGCATGTATACAGTAGGAGATTA | A fragment of KdcA used to construct pBAD-K |
| K2 | CGCCAAAACAGCCAAGCTTCGAATTCTCATGATTTATTTTGTTCAG |  |
| B1 | CATCATCATCATCATGGTATGGCTAGCATGACCCATCAATTAAGATCGC | A fragment of BrnQ used to construct pBAD-B |
| B2 | CGCCAAAACAGCCAAGCTTCGAATTCTTAGTGAGCGCTGGAGGTC |  |
| P1 | ATCATCATCATCATGGTATGGCTAGCATGGCACATCCACCACGGCT | A fragment of prpR used to construct pBAD-P |
| P2 | CGCCAAAACAGCCAAGCTTCGAATTCTCAGCTTTTCAGCCGCCGCC |  |
| KB1 | ATCATCATCATCATGGTATGGCTAGCATGTATACAGTAGGAGATTA | A fragment of KdcA used to construct pBAD-KB |
| KB2 | TCATGATTTATTTTGTTCAG |  |
| KB3 | CTGAACAAAATAAATCATGAAAGAAGGAGATATACCATGACCCATCAATTAAGATC | A fragment of BrnQ used to construct pBAD-KB |
| KB4 | CGCCAAAACAGCCAAGCTTCGAATTCTTAGTGAGCGCTGGAGGTC |  |
| KBP1 | ATCATCATCATCATGGTATGGCTAGCATGTATACAGTAGGAGATTAC | A fragment of KdcA used to construct pBAD-KBP |
| KBP2 | TGATTTATTTTGTTCAGCAA |  |
| KBP3 | TTGCTGAACAAAATAAATCACATCATCATCATCATCATTGAAAGAAGGAGATATACCATGACCCATCAATTAAGATCGCG | A fragment of BrnQ used to construct pBAD-KBP |
| KBP4 | AGCCGTGGTGGATGTGCCATGGTATATCTCCTTCTTTTAATGATGATGATGATGGTGAGCGCTGGAGGTCACCT |  |
| KBP5 | ATGGCACATCCACCACGGCT | A fragment of prpR used to construct pBAD-KBP |
| KBP6 | CGCCAAAACAGCCAAGCTTCGAATTCTCAATGATGATGATGATGATGGCTTTTCAGCCGCCGCCAGA |  |
| BpA1 | ATCATCATCATCATGGTATGGCTAGCATGACCCATCAATTAAGATCGC | A fragment of BrnQ used to construct pBAD-BpA |
| BpA2 | GTGAGCGCTGGAGGTCACCT |  |
| BpA3 | AGGTGACCTCCAGCGCTCACCATCATCATCATCATTAAAAGAAGGAGATATACCATGCTCTGTTTTGGAGTGAT | A fragment of FeS used to construct pBAD-BpA |
| BpA4 | ATTAAAACTTTTTCACCCATGGTATATCTCCTTCTTTTAATGATGATGATGATGATGCCTCCAAGGTGGTTTCATG |  |
| BpA5 | ATGGGTGAAAAAGTTTTAATGAAGG | A fragment of porA used to construct pBAD-BpA |
| BpA6 | CGCCAAAACAGCCAAGCTTCGAATTCTTAATGATGATGATGATGATGTTTTACGTATTCCTCTCCAGCT |  |
| L1 | ATCATCATCATCATGGTATGGCTAGCatgactaaaaaaatttcattcatt | A fragment of LuxCDABE used to construct pBAD-LuxCDABE |
| L2 | CGCCAAAACAGCCAAGCTTCGAATTCtcaactatcaaacgcttcg |  |
| ΔilvC1 | gtcctaggtataatactagtGTACGTGAAGAGTACAAACGgttttagagctagaaatagc | A fragment of ilvC-sgRNA used to construct pTargetT-ΔilvC |
| ΔilvC2 | ACAACGTCACATTGCAATTTctgcaggtcgactctagaga |  |
| ΔilvC3 | AAATTGCAATGTGACGTTGTGAATA | A fragment of ilvC-upstream used to construct pTargetT-ΔilvC |
| ΔilvC4 | TTTCATCAGTGGCTGTACGGT |  |
| ΔilvC5 | ACCGTACAGCCACTGATGAAATTCTGGAATCCTCTTTCGTT | A fragment of ilvC-downstream used to construct pTargetT-ΔilvC |
| ΔilvC6 | agggtaatagatctaagcttGGAATCAACCATGGTTTCAA |  |
| ΔilvI1 | gtcctaggtataatactagtCTGCTGATATCCCGATTGTGgttttagagctagaaatagc | A fragment of ilvI-sgRNA used to construct pTargetT-ΔilvI |
| ΔilvI2 | AATCCCCACCATGTCGCACTctgcaggtcgactctagaga |  |
| ΔilvI3 | AGTGCGACATGGTGGGGATT | A fragment of ilvI-upstream used to construct pTargetT-ΔilvI |
| ΔilvI4 | TACCCCGACGGCGAAAATCA |  |
| ΔilvI5 | TGATTTTCGCCGTCGGGGTAAGTGGCGCGCTCGTCA | A fragment of ilvI-downstream used to construct pTargetT-ΔilvI |
| ΔilvI6 | agggtaatagatctaagcttTTGCGGATGAGAGATCTGGATCCC |  |
| ΔLrp1 | gtcctaggtataatactagtGTCGAGCTTTCTAAACGTGTgttttagagctagaaatagc | A fragment of lrp-sgRNA used to construct pTargetT-Δlrp |
| ΔLrp2 | ACGTCTGTAAATTCCCTACActgcaggtcgactctagaga |  |
| ΔLrp3 | TGTAGGGAATTTACAGACGT | A fragment of lrp-upstream used to construct pTargetT-Δlrp |
| ΔLrp4 | GTCTCTCTGTATTCCTTCCC |  |
| ΔLrp5 | GGGAAGGAATACAGAGAGACTCACTTCTGGTATTCGTTGA | A fragment of lrp-downstream used to construct pTargetT-Δlrp |
| ΔLrp6 | agggtaatagatctaagcttAAGGATCAGCAACGCCTCCA |  |

**Table S4.** Primers for gene editing verification.

| prime name | sequence 5'-3' |
| --- | --- |
| △ilvI-Ver.F | GGAGATGTTGTCTGGAGCCGAGATG |
| △ilvI-Ver.R | CGCGAATCTGCATCGGGTAGACG |
| △ilvC-ver.F | CTTCCGTCAGCGTTACCGTG |
| △ilvC-ver.R | CCGTACTCGGCAGTATCGGAG |
| △Lrp-Ver.F | AGATCCCCATAGTTGTTGGCAGAC |
| △Lrp-Ver.R | GGGTTAAAGCTTAGTAAGGCAGCCATC |

**Table S5.** Primers for qPCR.

| prime name | sequence 5'-3' | product |
| --- | --- | --- |
| rpoA.F | ACTCTGGGTAACGCACTGCG | *rpoA of genome* |
| rpoA.R | CTGCAGTCACAGGGCCAATG |  |
| BrnQ-G.F | ACAACACCACATCCACAGGC | *BrnQ of genome* |
| BrnQ-G.R | CTTTTGCCAGCGCCACTACC |  |
| prpR-G.F | CGGTTTCTTAACTTTGCGGTGC | *prpR of genome* |
| prpR-G.R | TTAGAGCCAGCGGCAATGATG |  |
| K-B.P.F | CATTTGGAGCAACAGAAGATCGAGT | the link sequence between *KcdA* and *BrnQ* on plasmid |
| K-B.P.R | AACGGCAGTAATGAGGAAGCC |  |
| B-P.P.F | TTTACCGCTGGCCGAACAAG | the link sequence between *BrnQ* and *prpR* on plasmid |
| B-P.P.R | TCTTGCGGATGTAGGTCACTGC |  |
| por.F | GCAATCCAAGCAGGATGTGAATG | *por of plasmid* |
| por.R | CCTAATCCTGGACCTCCTCTAACG |  |
